# Knowledge, Beliefs and Attitudes of Patients and the General Public towards the Interactions of Physicians with the Pharmaceutical and the Device Industry: A Systematic Review

**DOI:** 10.1371/journal.pone.0160540

**Published:** 2016-08-24

**Authors:** Racha Fadlallah, Hala Nas, Dana Naamani, Fadi El-Jardali, Ihsan Hammoura, Lina Al-Khaled, Hneine Brax, Lara Kahale, Elie A. Akl

**Affiliations:** 1 Department of Health Management and Policy, Faculty of Health Sciences, American University of Beirut, Beirut, Lebanon; 2 Center for Systematic Reviews of Health Policy and Systems Research (SPARK), American University of Beirut, Beirut, Lebanon; 3 Faculty of Medicine, University of Damascus, Damascus, Syria; 4 Department of Biology, Faculty of Art and Science, American University of Beirut, Beirut, Lebanon; 5 Department of Clinical Epidemiology and Biostatistics, McMaster University, Hamilton, ON, Canada; 6 Department of Pediatrics and Adolescent Medicine, Faculty of Medicine, American University of Beirut, Beirut, Lebanon; 7 Faculty of Medicine, Université Saint Joseph, Beirut, Lebanon; 8 Department of Internal Medicine, American University of Beirut, Beirut, Lebanon; University of California San Diego, UNITED STATES

## Abstract

**Objective:**

To systematically review the evidence on the knowledge, beliefs, and attitudes of patients and the general public towards the interactions of physicians with the pharmaceutical and the device industry.

**Methods:**

We included quantitative and qualitative studies addressing any type of interactions between physicians and the industry. We searched MEDLINE and EMBASE in August 2015. Two reviewers independently completed data selection, data extraction and assessment of methodological features. We summarized the findings narratively stratified by type of interaction, outcome and country.

**Results:**

Of the 11,902 identified citations, 20 studies met the eligibility criteria. Many studies failed to meet safeguards for protecting from bias. In studies focusing on physicians and the pharmaceutical industry, the percentages of participants reporting awareness was higher for office-use gifts relative to personal gifts. Also, participants were more accepting of educational and office-use gifts compared to personal gifts. The findings were heterogeneous for the perceived effects of physician-industry interactions on prescribing behavior, quality and cost of care. Generally, participants supported physicians’ disclosure of interactions through easy-to-read printed documents and verbally. In studies focusing on surgeons and device manufacturers, the majority of patients felt their care would improve or not be affected if surgeons interacted with the device industry. Also, they felt surgeons would make the best choices for their health, regardless of financial relationship with the industry. Participants generally supported regulation of surgeon-industry interactions, preferably through professional rather than governmental bodies.

**Conclusion:**

The awareness of participants was low for physicians’ receipt of personal gifts. Participants also reported greater acceptability and fewer perceived influence for office-use gifts compared to personal gifts. Overall, there appears to be lower awareness, less concern and more acceptance of surgeon-device industry interactions relative to physician-pharmaceutical industry interactions. We discuss the implications of the findings at the patient, provider, organizational, and systems level.

## Introduction

Expenditure on pharmaceutical promotional activities has been on the rise globally, reaching $90 billion in 2012.[1] The amount of spending on drug promotion has reached $28 billion in the United States (USA) alone, $26 billion in Japan and $20 billion in five European countries (UK, France, Germany, Spain, and Italy).[1] Research contracts for clinical trials in the USA are now worth over $11 billion per year.[2] Moreover, pharmaceutical and medical device industries in the USA fund up to 60% of accredited continuing medical education costs.[3]

Physician interaction with the pharmaceutical and device industry can take several forms. These interactions include detailing (which are direct visits from pharmaceutical representatives to physicians to provide information about their company's drugs), industry gift-giving, distribution of free drug samples, and industry-sponsored meals. In addition, physicians take part in or lead industry-funded research, receive royalties for recommending or using industry devices, provide consultancy services, sit on advisory boards, deliver industry-developed presentations, and attend industry-funded continuing medical education.[4–7]

Interactions between physicians and the industry carry a number of advantages. One study found that pharmaceutical representatives provide information about medication indications and dosages to a relatively high percentage of physicians.[8] Similarly, interaction with the device industry has contributed to important technological and medical care advances.[9, 10] It has also allowed physicians to become more familiar with, and use the latest technology when caring for their patients, which has led to beneficial effects on patient outcome.[11, 12]

Nonetheless, the interactions between physicians and the industry remain controversial [4, 13] with concerns that such interactions may create conflicts of interest (COI). [14] Indeed, the evidence from several systematic reviews, albeit mostly from high income countries (USA, Canada, Australia, Denmark, Netherlands, New Zealand, France, Spain, Belgium, UK and Turkey) suggests an impact of physician-pharmaceutical industry interactions on increased prescribing frequency in favor of the promoted drugs, lower prescribing quality, and unnecessarily increased costs, which may have negative implications on patient care. [15–18]. Similarly, there have been reports of unethical financial relationships between surgeons and the device industry. [19–21] These included contracts paying royalties without any actual transfer of intellectual property, consulting agreements for minimal work, expensive meals disguised as medical lectures, payments for continuing medical education at expensive resorts, inappropriate gifts of no relevance to medicine such as laptop for home and direct payments to surgeons for utilizing specific implants.[22] This has raised concerns about surgeon-device industry interactions and conflicts of interest they can create.[20, 23–29] There are also concerns that such interactions can increase the costs of surgical devices, undermine patient-surgeon relationship, create public mistrust in medical institutions, and lead to inappropriate patient care. [30–32]

As a response, regulatory and advisory bodies in the USA, Canada and Australia have established guidelines for identifying, disclosing and managing potential COI. [32–34] The USA has recently implemented the Physician Payment Sunshine Provision Act which mandates the disclosure of payments from the pharmaceutical industry or device manufacturers exceeding $100 to federal authorities and publicly searchable web sites.[35] The American Academy of Orthopaedic Surgeons (AAOS) has proactively recommended direct disclosure to patients of any financial interaction with a manufacturer concerning the patient’s treatment. [36] The Australian Pharmaceutical Manufacturers Association has recently revised its code of conduct which now requires member pharmaceutical companies to report on payments made to individual health professionals for their services, educational grants, or sponsorships to attend educational events. Starting October 2016, public reporting of such payments will become mandatory. [37]

Understanding the patients’ and the general public’s perceptions of physician-industry interaction is necessary for informing policies and designing appropriate interventions.[38] The most recently conducted systematic review on this topic reviewed articles published between 1988 and 2009.[39] In addition, we are aware of at least five studies published since then, which can capture more current perceptions, particularly in light of the negative media attention that the pharmaceutical industry has received in recent years.[40–42]

The objective of this study was to systematically review the evidence on the knowledge, beliefs, and attitudes of patients and the general public towards the interactions of physicians with the pharmaceutical and the device industry. While this is similar to the objectives of prior studies in addressing pharmaceutical industry [39, 43], it also specifically examines the interactions between surgeons and the device industry.

## Methods

### Review protocol

A review protocol does not exist for this systematic review. However, we followed the methodology used for a related systematic review on interventions addressing physicians’ interactions with the pharmaceutical industry.[38]

### Eligibility criteria

We used the following inclusion criteria:

- Types of studies: quantitative studies (e.g. survey designs) and qualitative studies (e.g., interviews, focus groups);
- Types of participants: patients and the general public;
- Types of exposure: any type of interaction between physicians and the pharmaceutical or device industry (e.g. meeting with drug representatives or medical/surgical device manufacturers; pharmaceutical-sponsored continuing medical education including travel funding; receiving free drug samples; industry-provided meals; gifts; financial interactions; consultancy);
- Types of outcomes: knowledge, beliefs, or attitudes of patients or the general public regarding physician-industry interactions and regulations of such interactions. We used the following classification [44] to distinguish between “attitudes”, “beliefs” and “knowledge”:
  ○ Knowledge of patients and the general public (e.g. towards the existence and extent of physician-industry interactions);
  ○ Beliefs of patients and public (e.g. towards the effects of such interaction on clinical practice and cost);
  ○ Attitudes of patients and public (e.g. towards acceptability and appropriateness as well as regulations of such interactions).

We excluded papers that:

- focus on interactions with residents or research investigators who are not acting as providers of clinical care;
- investigate the knowledge, beliefs, and/or attitudes of physicians or other healthcare professionals towards such interactions (this will be addressed in another systematic review);
- assess the effects of financial ties on enrolment into research.

We also excluded opinion polls, editorials, letters to the editor, systematic reviews and non-English studies. We did not exclude studies based on date of publication.

### Search strategy

We used OVID interface to electronically search MEDLINE and EMBASE in August 2015. The search included both free text words and medical subject heading. It combined terms for physicians and pharmaceutical and did not use any search filter. A medical librarian helped with the design of the search strategy. The full details of the search strategy are provided in S1 Appendix. We also searched the grey literature and reviewed the references lists of included and relevant papers.

### Selection of studies

Teams of two reviewers (RF, HN, DN, and IH) screened the titles and abstracts of identified citations in duplicate and independently for potential eligibility. We retrieved the full text of citations considered as potentially eligible by at least one of the two reviewers. The same teams of reviewers then screened the full texts in duplicate and independently for eligibility. They conducted calibration exercises and used a standardized and pilot tested screening form. They resolved any disagreement by discussion or with the help of a third reviewer.

### Data collection

Teams of two reviewers (RF, HN, DN, and IH) abstracted data from eligible studies in duplicate and independently. They conducted calibration exercises and used a standardized and pilot tested screening form adapted from a study conducted by Akl et al., 2013.[45] If disagreements arose, they were resolved through discussion or with the input of a third reviewer. We collected data on the following:

1. Funding source, type of interaction;
2. Methodological quality: sample size calculation, sample frame, sampling method, recruitment and administration method, response rate, validity of survey tool, and pilot testing;
3. Participants: country, participant characteristics, setting, numbers of participants;
4. Results: knowledge, beliefs and attitudes towards physicians’ interaction with the industry, regulations of such interactions.

### Assessment of risk of bias in included studies

Teams of two reviewers (RF, HN, DN, and IH) assessed the risk of bias in each eligible study in duplicate and independently and resolved any disagreement by discussion or with the help of a third reviewer. They used the Critical Appraisal Skills Program (CASP) tool to assess the risk of bias in qualitative studies.[46] They assessed the methodological quality of surveys using the above listed methodological quality criteria.

### Data analysis and synthesis

We calculated the kappa statistic to assess the agreement between reviewers assessing full texts for eligibility. Two authors (EAA, RF) reviewed the results of individual studies to identify common themes and develop a structure for reporting the results. We did not conduct a meta-analysis due to the nature of the data. Instead, we reported the findings in a narrative way, stratified by type of interaction (pharmaceutical vs. device industry), type of outcome and country of the study. We also indicated the year of the study for each finding (and if not reported, the year of publication).

## Results

**Fig 1:**
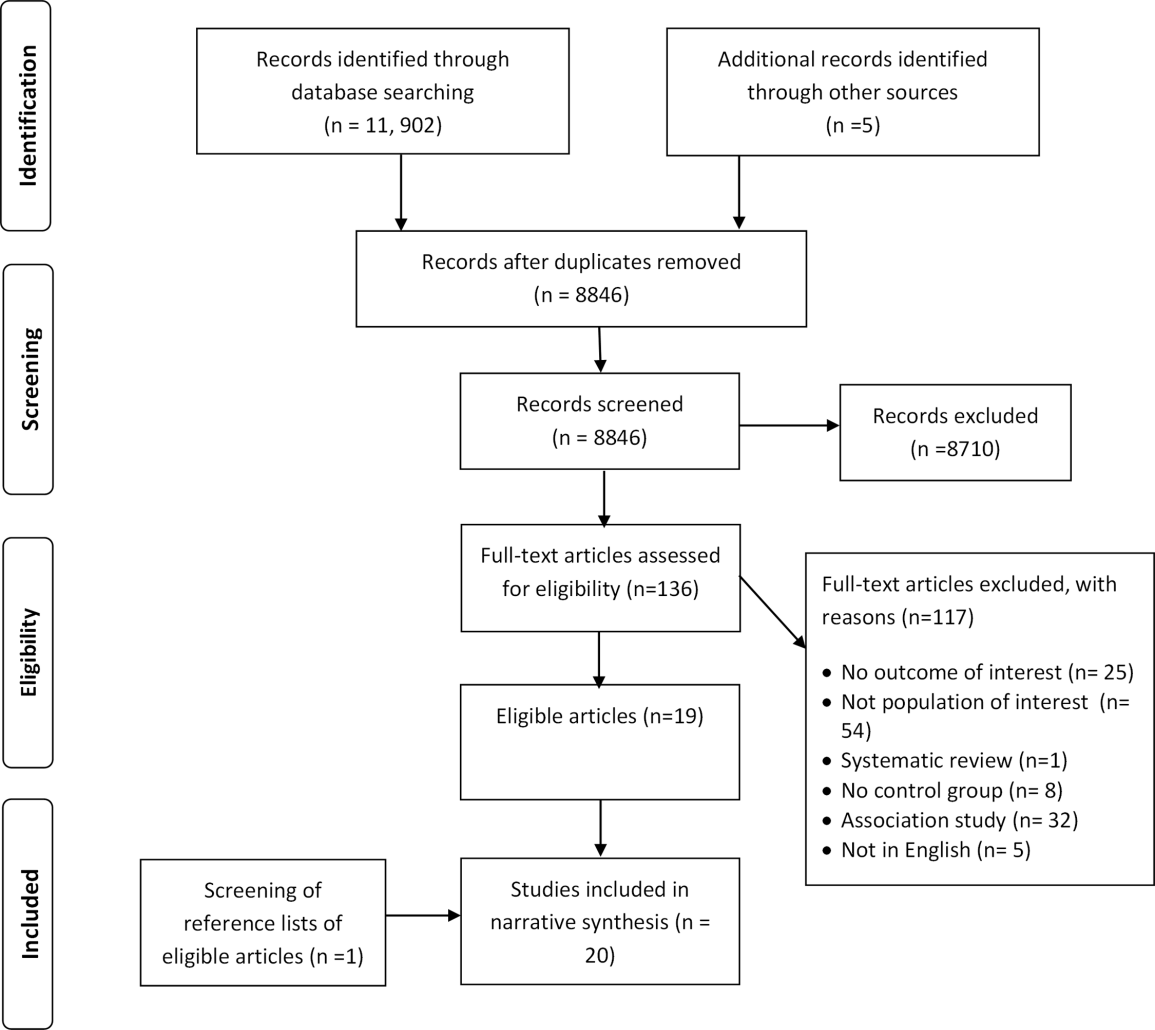
Study flowchart.

The process of literature search is displayed in the flowchart (Fig 1). Of the 11,902 citations identified through database and websites searches, 20 studies met our inclusion criteria. The publication years of the included studies ranged from 1995 to 2015. We excluded 117 studies for the following reasons: no outcome of interest (n = 25); no population of interest (n = 54); systematic review (as opposed to primary research) (n = 1); focus on association between industry interaction and clinical practice (n = 32); and not in English (n = 5) (See S2 Appendix for the list of excluded studies with reasons for exclusion). The kappa statistic value for full text screening was 0.89, suggesting high levels of agreement.

Of the twenty included studies, four focused on surgeons’ interactions with the device industry (e.g. consultancy relationships).[7, 47–49] Fifteen studies focused on the pharmaceutical industry and ranged from drug samples and office gifts to receiving personal gifts. The remaining study examined both types of interactions.[50] We report the results according to whether the relationship is with the pharmaceutical industry or with the device industry (parts 1 and 2 respectively).

### Part 1. Physician-pharmaceutical industry interactions

#### Characteristics of included studies

Sixteen studies examined the interactions between physicians and the pharmaceutical industry. The characteristics of each included study are described in Table 1. Countries in which the studies were conducted were: the USA (n = 8), Australia (n = 3), Canada (n = 1), Turkey (n = 1), Pakistan (n = 1), and South Africa (n = 1). One study included patients from both the USA and Canada.[50] Ten studies recruited patients from hospitals and clinics while six studies recruited individuals from the general public. Only one of the included studies was of qualitative nature and consisted of focus group sessions.[51]

**Table 1:** Characteristics of included studies on physicians’ interactions with the pharmaceutical industry.

Study ID	Sample size and funding	Participants and settings	Sampling frame and method	Type of interaction	Outcomes assessed
**Blake, 1995[52]**	• N = 486 • Funding not reported	• Adults (I8 years of age and older) in two family practice centers • Columbia, USA; June and July 1994 • 63.2% females; mean age (SD): 40.6 (±15.8) • Education: 48.4% some college or college graduate; 17.1% postgraduate degree	• Frame: Adults in two family practice centers operated by the University of Missouri-Columbia, Medicine. • Method: Convenience sampling	• Free drug samples • Ballpoint pens • Medical books • Baby formula • Dinner at a restaurant • A coffee maker	• Awareness of the interactions of physicians in general • Beliefs about their effects on prescription behavior and quality of care • Beliefs about their effects on cost of care • Attitudes towards the interactions
**La Puma, 1995[53]**	• N = 200 • Funding not reported	• Patients (18 years and above) in a general medical office • USA • 64% females; mean age (range): 49.7 (18 to 87 years)	• Frame: Patients 18 years and above in a general medical office. • Method: Convenience sampling	Financial payment for taking part in post marketing research for drugs	• Beliefs about their effects on prescription behavior and quality of care • Attitudes towards the interactions • Attitudes towards possible ways to manage the interactions: - Disclosure of interaction
**Mainous, 1995[54]**	• N = 649 • Funding not reported	• Kentucky residents (18 years of age and older) • Kentucky, USA • 55% females; mean age (SD): 47± 16 • Education: 72% high school or above	• Frame: Data base of phone numbers • Method: stratified random sampling	• Office use gifts (e.g., samples, pens, notepads) • Personal gifts (clocks, radios, or dinners at expensive restaurants)	• Awareness of the interactions of physicians in general • Beliefs about their effects on prescription behavior and quality of care • Beliefs about their effects on cost of care • Attitudes towards the interactions
**Gibbons, 1998[55]**	• N = 196 (100 at military site and 96 at civilian site) • Funding not reported	• Patients at two medical centers • USA • 65–67% female; mean age (range): 61 (21–89) at military site, 60 (24–90) at civilian site • Education: college graduate: 29% at military site; 3.2% at civilian site	• Frame: Patients at two tertiary-care medical centers, one military and one civilian, at Washington, DC. • Method: Random sampling at military center, convenience sampling at civilian center.	• Trip • Dinner • Pocketknife • Lunch • Mug • Drug sample • Large text; small text • Pen • Video	• Awareness of the interactions of own physicians • Awareness of the interactions of physicians in general • Beliefs about their effects on prescription behavior and quality of care • Attitudes towards the interactions
**Qidawai, 2003[56]**	• N = 420 • Funding not reported	• Patients attending outpatient tertiary care hospital • Pakistan; December 1999 to May 2000. • 11.2% females; mean age (SD): 33.7 (±11.98) • Education: 34% graduate	• Frame: Patients attending outpatient settings of a busy tertiary care hospital • Method: Convenience sampling	Accepting gifts from pharmaceutical companies	• Attitudes towards the interactions
**Semin, 2006[57]**	• N = 584 • Funded by a research grant from the Dokuz Eylul University, Turkey	• Patients admitted to the primary health care centers in Izmir Centrum • Turkey; December 2004 • 64.7% females; mean age (SD): 42 (± 15.4); 34.9% with chronic disease • Education: 21% university	• Frame: Patients who had been admitted to the primary health care centers in Izmir Centrum, the third largest city in Turkey. • Method: Stratified systematic sampling	• Obtaining medical devices for office • Invitation to the conferences for the week-end at hotels • Conference and dinner • Middle level gifts • Invitation for the congresses • Medical booksLow level gifts e.g. pen	• Awareness of the interactions of physicians in general • Beliefs about their effects on prescription behavior and quality of care • Beliefs about their effects on cost of care • Attitudes towards the interactions • Attitudes towards the effects of interactions on trust • Attitudes towards possible ways to manage the interactions: - Regulation of interaction
**Edwards, 2009[58]**	• N = 134 • Supported by Donaghue Initiative at Yale University Interdisciplinary Bioethics Center	• Employees of The Age newspaper in Melbourne • Australia; 18 January and 8 February 2007 • 57.8% female; age: 40% 31–43, 34.1% 18–30, 25.3% 44–65 • Education: 37.6% Bachelor’s degree; 14.5% postgraduate degree;	• Frame: employees of The Age newspaper in Melbourne. N = 1524 • Method: Convenience sampling	Pharmaceutical marketing activities ranging from largesse such as small gifts and free drug samples, to the sponsorship of educational conferences	• Awareness of the interactions of physicians in general • Beliefs about their effects on prescription behavior and quality of care • Attitudes towards the interactions • Attitudes towards the effects of interactions on trust • Attitudes towards possible ways to manage the interactions: - Disclosure of interaction - Method of disclosure
**Jastifer, 2009[59]**	• N = 903 • Supported by Upper Peninsula Health Education Corporation, Michigan State University	• Adult residents (18 years and older) who reside in Alger County • Michigan, USA • 63.1% females; age: 12.8% aged 18–40, 39.5% aged 41–60, 47.7% older than 60 • Education: 50.7% high-school graduate or some college; 34.8% college graduate or postgraduate degree	• Frame: List of postal addresses • Adult residents, 18 years and older, who reside in Alger County, in rural Michigan.” • Method: Convenience sampling	• Drug samples • Ballpoint pens • Medical books • Conference/travel expense • Dinner out • Spouse meal at dinner out • Golf tournament fees	• Awareness of the interactions of physicians in general • Beliefs about their effects on prescription behavior and quality of care • Beliefs about their effects on cost of care • Attitudes towards the interactions • Attitudes towards possible ways to manage the interactions: - Disclosure of interaction
**Tattersall, 2009[60]**	• N = 906 • Funding not reported	• Patients in the waiting rooms of three general practices • Australia; October to November 2007 • 48.5% female; mean age (SD): 51.2 (±104.7) • Education: 71.3% undergraduate or postgraduate university degree	• Frame: Three general practices in metropolitan Sydney • Method: Convenience sampling	• Benefits in cash or in kind • Financial incentives for participation in research activities • sponsor for travel • Registration or accommodation to attend conferences • Indirect benefit /financial incentive for instituting treatment course, prescribing a drug, making a referral, doing test or procedure, enrolling patients in trial	• Awareness of the interactions of own physicians • Beliefs about their effects on prescription behavior and quality of care • Attitudes towards possible ways to manage the interactions: - Disclosure of interaction - Method of disclosure
**Macneill, 2010[61]**	• N = 757 • Supported by National Health and Medical Research Council of Australia	• General public (over the age of 18 years) from the electoral roll of the Hunter region • New South Wales, Australia • 59% female; average age (SD): 52.2 (±16.2) • Education: 20% university degree or currently attending a university	• Frame: Electoral roll of the Hunter region of New South Wales • Adults of New South Wales over the age of 18 years • Method: Random sampling	• Gifts with potential • benefit to patients (e.g. leaflets, drug samples, appointment books, flashlight) • Office-use gift (e.g. • pens, Spirometer/ECG machine stethoscope, surgery computer) • Personal gift • (conference, ticket, laptop)	Attitudes towards the interactions
**Grande, 2012[62]**	• N = 2,029 • Funded by National Human Genome Research Institute, American Cancer Society	• Adults in 10 large metropolitan areas • USA; June -December 2006 • 63.2% female; age: 8.4% aged 18–39, 62.2% aged 40–64, 29.4% aged 65 and above • Education: 28.2% some college; 35.3% 4-year college degree or graduate school.	• Frame: A database of phone numbers • Method: Cluster random sampling	Pharmaceutical industry–physician gift relationships	• Awareness of the interactions of own physicians • Awareness of the interactions of physicians in general • Attitudes towards the effects of interactions on trust
**Green, 2012[63]**	• N = 192 • Funding not reported	• English-speaking adults in outpatient clinics waiting rooms • USA; 2008 • 61% female; mean age (range): 53 (18–89) • Education: 45% high school graduate or some college; 46% college graduate or more	• Frame: patients in waiting rooms of five outpatient clinics at a mid-Atlantic academic medical center. • Method: Convenience sampling	• Accepted large gifts • Attend drug company social activities and trips • Accepted small gifts • Gave lecture • Conducted research for drug company • Accepted industry-sponsored meals	• Awareness of the interactions of own physicians • Beliefs about their effects on prescription behavior and quality of care • Attitudes towards the interactions • Attitudes towards the effects of interactions on trust • Attitudes towards possible ways to manage the interactions: - Disclosure of interaction
**Wise, 2012[64]**	• N = 200 • Funding not reported	• Postoperative South African patients from four surgical wards in a teaching hospital • South Africa; March- Nov 2011 • 67% females; age: 17% aged 18–24, 73% aged 25–64, 10% 65 or above • Education: Not reported	• Frame: Postoperative adult patients at Grey’s Hospital, Pietermaritzburg. • Method: Convenience sampling	• Samples; • Small gifts (e.g. pens, notepads) • Fees for speaking at industry -sponsored conferences • Free food and dinners • Travel or holidays	• Beliefs about their effects on prescriptive behavior and quality of care • Attitudes towards the interactions • Attitudes towards the effects of interactions on trust • Attitudes towards possible ways to manage the interactions: - Disclosure of interaction
**Camp, 2013[50]**	• N = 503 • No external funding sources	• Postoperative arthroplasty patients attending follow up hip and knee arthroplasty clinics • USA & Canada; Nov 2010-March 2011 • 55% females US; 59% females Canada; age: 36% < 60, 64% 60 and above for US; 30% < 60, 69% 60 and above for Canada • Education: US (51% college /university degree; Canada (51% college/university degree	• Frame: postoperative patients attending follow up hip and knee arthroplasty clinics at Mount Sinai Hospital and Holland Orthopaedic • Method: convenience sampling	Financial relationships with manufacturers (gifts, royalties, consultancy payments, speakers’ bureau presentations, or research support)	Awareness of the interactions of physicians in general
**Holbrook, 2013[65]**	• N = 1041 • Funded by Canadian Institutes of Health Research	• Adult population (18 years of age or older) who speak English or French and reside in private homes • Canada; May-September 2010 • 56.8% female; mean age (SD): 52.6 (16.5) • Education: 57.7% college or higher	• Frame: A database of phone numbers Method: Stratified random sampling.	• Requesting information about a particular drug • Educational gifts to patient • Free meals to listen to industry personnel • Payment to attend conference • Research recruitment fees • Medication samples	Attitudes towards the interactions
**Oakes, 2015[51]**	• N = 31 (a total of six focus groups) • Funding not reported	• Patients from three of the academic health center’s clinics (orthopedic surgery, cardiology and dentistry) • USA, Twin Cities area Minnesota; nine-week period (no data) • 74% female; mean age: 55 • Education: 65% college	• Frame: Participants 18 years and older from three clinics (orthopedic surgery, cardiology and dentistry) in one academic health center • Method: convenience sampling	Conflict of interest relating to physician interaction with the industry	• Attitudes towards the interaction • Attitudes towards the effects of interactions on trust • Attitudes towards possible ways to manage the interactions: - Disclosure of interaction - Methods of disclosure

The included studies assessed the following types of outcomes: knowledge (n = 10), beliefs (n = 10), and attitudes (n = 14) of patients and the general public regarding the interactions of physicians with the pharmaceutical industry. Eight studies also examined the attitudes of patients and the general public towards possible ways to manage those interactions (e.g. disclosure and regulation of physician-industry interactions). Most of the studies assessed more than one type of outcome.

#### Methodological features

The methodological features of the 16 included studies are described in S1 Table.

While all sixteen studies examining the relationship with the pharmaceutical industry described their sampling frame, fewer fulfilled the following factors related to the risk of bias: reporting sample size calculation (n = 2) [57, 60]; using random approach to sampling (n = 6) [54, 55, 57, 61, 62, 65]; reporting using validated tools (n = 4) [53, 62, 63, 65]; reporting pilot-testing the tools (n = 7). [7, 50, 52, 53, 55, 58, 60, 63] The response rate was not reported for three studies [49, 57, 64] and varied across the remaining from 8.8% to 96%. The single qualitative study was judged to have met most of the CASP checklist.

#### Summary of findings

We provide a detailed summary of the findings for each included study in S2 Table. We also provide below a narrative synthesis of the results organized according to the outcomes of interest described under the eligibility criteria section above:

- Awareness of the interactions between own physician and the pharmaceutical industry (n = 4 studies)
- Awareness of the interactions between physicians in general and the pharmaceutical industry (n = 7)
- Beliefs about the effects of interactions on prescription behavior and/or quality of care (n = 10)
- Beliefs about the effects of interactions on cost of care (n = 5)
- Attitudes towards the interactions (n = 10)
- Attitudes towards the effect of interactions on trust (n = 6)
- Attitudes towards possible ways to manage the interactions (n = 8)

Awareness of the interactions between own physician and the pharmaceutical industry (n = 4): The four studies were conducted in the United States (n = 3) and Australia (n = 1). The majority of patients in the four studies reported being unaware of possible interactions between their own physicians and the pharmaceutical industry.

The findings from the three studies conducted in the USA are shown below:

- 27% out of 196 patients at two medical centers in 1998 thought their own physician accepted gifts (20% responded ‘no’ and 53% ‘were unsure’).[55]
- In a survey of 192 adults admitted to outpatient clinics in 2008, 3% to12% were aware that their physicians could accept gifts greater than $100 in value, attend industry-sponsored social activities, go on trips paid for by the industry, accept gifts less than $100, give lectures for drug companies, conduct research for drug companies, or accept drug company meals.[63]
- In the study with qualitative data published in 2015, only three patients (out of 31) recruited from academic health center’s clinics stated that they had been told by a physician about a conflict of interest; however, the latter was described as ‘uneventful’ and not having an impact on the care provided or the patient-provider relationship.[51]

The study conducted in Australia found that 12% out of 906 patients in the waiting rooms of three general practices in 2007 were aware that their doctor may have a competing interest with drug companies (76% were unaware).[60]

Awareness of the interactions between physicians in general and the pharmaceutical industry (n = 7): Of the seven included studies, four focused on gifts in general and three focused on specific types of gifts.

The studies focusing on gifts in general were conducted in the USA (n = 2), Australia (n = 1), and Turkey (n = 1). The majority of participants in these studies reported being aware of pharmaceutical promotional activities in general (see Table 2). Below, we present the findings for each study:

- 54% out of 196 patients at two medical centers in the USA in 1998 were aware of gifts given to physicians. Among those who were unaware, 24% said that such knowledge altered their perception of the profession.[55]
- 70% out of 251 patients in the USA and 55% out of 252 in Canada attending post-operative clinics between 2010 and 2011 were aware of physicians’ interactions with pharmaceutical companies.[50]
- 83% out of 584 patients admitted to primary health care centers in Turkey in 2004 were aware of pharmaceutical promotional activities.[57]
- 40% out of 134 employees sampled from *The Age newspaper* in Australia in 2007 were aware of pharmaceutical marketing.[58]

**Table 2:** Patient and public awareness of gifts received by physicians in general from the pharmaceutical industry.

Awareness of gifts received by physicians from the pharmaceutical industry			
Type of Gifts	% aware (N)^1^	Country, year^2^	Reference
Gifts in general (pharmaceutical promotional activities/ pharmaceutical marketing)	54% (N = 196)	USA, 1998	[55]
	70% (N = 503)	USA, 2010–2011	[50]
	55% (N = 252)	Canada, 2010–2011	[50]
	70% (N = 251)	USA, 2010–2011	[50]
	40% (N = 134)	Australia, 2007	[58]
	83% (N = 584)	Turkey, 2004	[57]
Gifts with possible patient benefit	82% (N = 649)	USA, 1995	[54]
Personal gifts (e.g. clocks, radios)	32% (N = 649)	USA, 1995	[54]
Drug samples	87% (N = 486)	USA, 1995	[52]
	94% (N = 903)	USA, 2009	[59]
Ballpoint pens	55% (N = 489)	USA, 1995	[52]
	76% (N = 903)	USA, 2009	[59]
Medical books	35% (N = 486)	USA, 1995	[52]
	38% (N = 903)	USA, 2009	[59]
Dinner	22% (N = 486)	USA, 1995	[52]
	37% (N = 903)	USA, 2008	[59]
Attend industry-sponsored trips/conferences/social activities	16–17% (N = 192)	USA, 2008	[63]
	34% (N = 903)	USA, 2009	[59]
Accept gifts over $100	12% (N = 192)	USA, 2008	[63]
Accept gifts less than $100	16% (N = 192)	USA, 2008	[63]
Conduct research for drug company	23% (N = 192)	USA, 2008	[63]
Baby formula	29% (N = 486)	USA, 1994	[52]
Coffee maker	14% (N = 486)	USA, 1994	[52]

^1^ N refers to the sample size

^2^ This indicates year of the study for each finding (and if not reported, the year of publication)

The three studies focusing on specific types of gifts were all conducted in the USA. Overall, greater awareness was reported for office-use gifts and gifts of potential benefits to patients relative to personal gifts. In one study conducted in 1994, only 32% out of 649 respondents from the general public were aware that physicians received personal gifts (e.g., clocks, radios, or dinners at expensive restaurants), whereas the majority (82%) were aware that physicians received office-use gifts (e.g. samples of medicine, pens, and pads of paper).[54] In another study conducted in 1994, the rate of awareness among 486 individuals attending family practice centers varied by type of gift as follows: drug samples (87%); ballpoint pens (55%); medical books (35%); dinner at a restaurant (22%); baby formula (29%); and coffee maker (14%).[52] The third study conducted in 2009 used a similar set of questions as the preceding study, with the following reported rates of awareness among 903 members of the general public: drug samples (94%); ballpoint pens (76%); medical books (38%); dinner out (37%); conference/travel expenses (34%); and golf tournament fees (19%)[59].

Beliefs about the effects of interactions on prescription behavior and/or quality of care (n = 10): The ten studies assessing the interactions of physicians with the pharmaceutical industry were conducted in the United States (n = 6), Australia (n = 2), Pakistan (n = 1), and South Africa (n = 1). Heterogeneity was observed across studies in the perceived effects of physician-pharmaceutical industry interactions on prescribing behaviors and quality of care. In five studies, less than half of the participants believed that gifts to physicians affected prescription behavior and/or quality of care (see Table 3).

**Table 3:** Beliefs of patients and the general public about the effects of physician-pharmaceutical industry interaction on prescribing behavior, quality of care, and cost of care.

**Beliefs about the effects of interaction on prescribing behavior**							
**Type of interaction**		**Influences prescribing behavior**		**Has little or no influence on prescribing behavior**	**Not sure/Don’t know**	**No opinion/ Neutral**	
	**Country, year**^**1**^	**% (N)**^**2**^		**% (N)**	**% (N)**	**% (N)**	**Reference**
**Gifts in General**	USA, 1995	70% (N = 486)		24.5% (N = 486)	-	-	[52]
	USA, 2009	41.2% (N = 903)		16.9% (N = 903)	32.8% (N = 903)	9.1% (N = 903)	[59]
	USA, 2008	49% (N = 192)		-	-	-	[63]
	Australia, 2007	59% (N = 134)		-	-	-	[58]
	Turkey, 2004	29% (N = 584)		37% (N = 584)	33% (N = 584)	-	[57]
	Australia, 2007	-		49% (N = 906)	-	24% (N = 906)	[60]
**Trip/Travel**	Turkey, 2004	69.9% (N = 584)		6.8% (N = 584)	23.3% (N = 584)	-	[57]
	USA, 1998	56% (N = 196)		-	-	-	[55]
**Dinner**	Turkey, 2004	62.8% (N = 584)		14.3% (N = 584)	22.9% (N = 584)	-	[57]
	USA, 1998	48% (N = 196)		-	-	-	[55]
**Drug sample and medical books**	Turkey, 2004	46.1% (N = 584)		33.2% (N = 584)	20.7% (N = 584)	-	[57]
	USA, 1998	42% (N = 196)		-	-	-	[55]
**Pen**	USA, 1998	31% (N = 196)		-	-	-	[55]
	Turkey, 2004	32.6% (N = 584)		52.6% (N = 584)	14.8% (N = 584)	-	[57]
**Obtaining an electrocardiogram or medical devices for the office**	Turkey, 2004	71–74% (N = 584)		5.1–9.8% (N = 584)	19.3–20.8% (N = 584)	-	[57]
**Invitation to conferences for the weekend at hotels /invitation for the congresses**	Turkey, 2004	59.2–63.5% (N = 584)		8.9–19.5% (N = 584)	27.6–29.8% (N = 584)	-	[57]
**Cover for the car seats**	Turkey, 2004	61.7% (N = 584)		19.7% (N = 584)	18.6% (N = 584)	-	[57]
**Pocketknife**	USA, 1998	28% (N = 196)		-	-	-	[55]
**Beliefs about the effect of interaction on quality of care**							
			**Influences decision/care**	**May influence decision/care**	**Has little or no influence**	**Not sure/No opinion**	
	**Country, year**		**% (N)**	**% (N)**	**% (N)**	**% (N)**	**Reference**
**Gifts in General**	Australia, 2007		27% (N = 906)	**-**	49% (N = 906	24% (N = 906)	[60]
	South Africa, 2011		80% (N = 200)	14% (N = 200)	7% (N = 200)	-	[64]
			**Positive effect**	**No effect**	**Negative effect**	**Don’t know**	
**Office use gifts**	USA, 1995		14% (N = 649)	61% (N = 649)	13% (N = 649)	12% (N = 649)	[54]
**Personal gifts**	USA, 1995		8% (N = 649)	54% (N = 649)	23% (N = 649)	15% (N = 649)	[54]
**Beliefs about the effect of interaction on cost of care**							
			**Increases cost**	**No effect**	**Decreases cost**	**Don’t know**	
	**Country, year**		**% (N)**	**% (N)**	**% (N)**	**% (N)**	**Reference**
**Gifts in General**	USA, 1995		64% (N = 486)	23% (N = 486)	3.1% (N = 486)	-	[52]
	USA, 2009		67.3% (N = 903)	31.6% (N = 903)	1.2% (N = 903)	-	[59]
	USA, 1998		33% (N = 196)	39% (N = 196)	-	-	[55]
	Turkey, 2004		54.5% (N = 584)	-	-	35.2% (N = 584)	[57]
**Office-use gift**	USA, 1995		26% (N = 649)	38% (N = 649)	19% (N = 649)	16% (N = 649)	[54]
**Personal gift**	USA, 1995		42% (N = 649)	30% (N = 649)	14% (N = 649)	14% (N = 649)	[54]

^1^ This indicates year of the study for each finding (and if not reported, the year of publication)

^2^ N refers to the sample size

The findings from the six studies conducted in the USA varied as follows:

- 70% out of 486 participants in the waiting rooms of two family practice centers in 1994 believed that gifts ‘sometimes’ or ‘frequently’ influenced a physician's prescribing of medication.[52]
- 13% out of 649 adult residents in a study conducted in 1995 believed that office-use gifts had a negative effect and 14% believed they had a positive effect on quality of care (as opposed to ‘no effect’ or ‘don't know’). The corresponding percentages for the effects of personal gifts were 23% and 8%, respectively.[54]
- 69% out of 200 patients visiting a general medical office in 1995 thought that some doctors might be influenced to enroll patients in research just for the fee.[53]
- In a survey of 196 patients admitted to two medical centers in 1998, the following gifts were most frequently perceived to influence prescription: trip (56%), dinner (48%) and drug sample (42%). The following gifts were least frequently perceived to influence prescription: pen (31%), lunch (29%) and pocketknife (28%).[55]
- 49% out of 192 participants in outpatient clinics in 2008 agreed that gifts or meals influenced physician’s prescribing behaviors. Furthermore, 43% believed that physicians who accepted small gifts in return for listening to a pharmaceutical representative’s presentation on a particular drug would be more likely to prescribe that medication. [63]
- 41% out of 903 members of the general public in 2009 believed that receiving a gift from a drug company influenced a physician’s prescribing behavior (17% said ‘no’; 33% said ‘maybe’; and 9% said ‘no opinion’).[59]

The two studies conducted in Australia in 2007 reported the following results:

- 49% out of 906 patients in the waiting rooms of three general practices believed that doctors were ‘not unduly influenced’ despite receiving benefits or perks (27% disagreed and 24% stated they were unsure).[60]
- 59% out of 134 employees of The Age newspaper in Melbourne rated a high level of agreement with the statement that “pharmaceutical companies influenced doctors’ prescriptions”.[58]

Two studies were conducted in low- and middle-income countries (LMICs) with the following results:

- 29% out of 584 patients admitted to primary health care centers in Turkey in 2004 believed that physicians made their drug choices according to the gifts and advertisements of pharmaceutical companies (37% answered ‘never’ and 33% said they ‘didn’t know’). Additionally, 51% to 68% agreed that receiving medical devices for office use, travels and invitations to conferences for the weekend at hotels had strong effects on prescriptions. On the other hand, medical books and pens were considered to have a strong effect on prescriptions by a small percentage of respondents (22% and 9% respectively).[57]
- 80% out of 200 postoperative patients in South Africa in 2011 believed that doctors were influenced by gifts from the pharmaceutical company.[64]

Beliefs about the effects of interactions on cost of care (n = 5): We identified five eligible studies: 4 conducted in the USA [52, 54, 55, 59] and 1 in Turkey (see Table 3).[57] The majority of participants in three of the five studies believed that gifts to physicians increased the cost of medication.

The four studies conducted in the USA reported the following results:

- 64% out of 486 adults in the waiting rooms of two family practices in 1995 believed that gifts to physicians increased the cost of medication.[52]
- 26% out of 649 members of the general public in a study conducted in 1995 believed that office-use gifts had negative effects on costs while 42% believed the same for personal gifts (38% and 30% respectively thought that gifts had no effect).[54]
- 33% out of 196 patients at two medical centers in 1998 believed that the cost of gifts was ultimately passed on to patients (39% disagreed and 28% were unsure).[55]
- 67.3% out of 903 members of the general public in 2009 believed that gifts to physicians increased the cost of medications (31.6% said ‘no effect,’ and 1.2% said it ‘decreases cost’).[59]

In the study conducted in Turkey in 2004, 55% out of 584 patients admitted to primary health care believed that promotion expenditures increased the cost of medications.[57]

Attitudes towards the interactions (n = 10): The ten studies were conducted in the USA (n = 5), Australia (n = 2), Pakistan (n = 1), Turkey (n = 1) and South Africa (n = 1). The attitudes varied by type of gifts across studies, with higher acceptance constantly reported for office-use gifts and gifts of potential benefits to patients as compared to personal gifts (see Table 4).

**Table 4:** Attitudes of patients and the general public towards physicians’ interactions with the pharmaceutical industry.

**Attitudes towards the appropriateness/acceptability of interaction**						
**Type of interaction**		**Can accept as much as offered**	**Shoud be limited to less than $25**	**Can accept between $25-$1000**	**Don’t know**	
	**Country, year**^**1**^	**% (N)**^**2**^	**% (N)**	**% (N)**	**% (N)**	**Reference**
**Office use gifts**	USA, 1995	59% (N = 649)	9% (N = 649)	12% (N = 649)	22% (N = 649)	[54]
**Personal gifts**	USA, 1995	33% (N = 649)	32% (N = 649)	14% (N = 649)	2% (N = 649)	[54]
		**Appropriate/ Approve**	**Not appropriate/ Do not approve**	**Don’t know**	**Neutral**	
	**Country, year**	**% (N)**	**% (N)**	**% (N)**	**% (N)**	**Reference**
**Gifts in general**	Pakistan, 1999–2000	88% (N = 420)	9% (N = 420)	3% (N = 420)	-	[56]
**Gift of little monetary value**	USA, 2008	43% (N = 192)	27% (N = 192)	-	30% (N = 192)	[63]
	South Africa, 2011	38% (N = 200)	62% (N = 200)	-	-	[64]
**Dinner**	USA, 1995	34.6% (N = 486)	48.4% (N = 486)	-	14.6% (N = 486)	[52]
	USA, 1998	-	47% (N = 196)	-	32.9% (N = 903)	[55]
	USA, 2009	12.1% (N = 903)	55% (N = 903)	-	-	[59]
	South Africa, 2011	12% (N = 200)	-	-	-	(64)
	Australia, 2010	34% (N = 757)	-	-	-	[61]
**Lunch (for doctor and staff)**	Australia, 2010	66–83% (N = 757)	-	-	-	[61]
**Pocket knife, mug**	USA, 1998	-	23–38% (N = 196)	-	-	[55]
**Drug sample**	USA, 1995	82% (N = 486)	8% (N = 486)	-	9% (N = 486)	[52]
	USA, 2009	69% (N = 903)	9% (903)	-	22% (N = 196)	[59]
	Australia, 2010	92% (N = 757)	-	-	-	[61]
	Turkey, 2004	83% (N = 584)	10% (N = 584)	7% (N = 584)	-	[57]
	South Africa, 2011	46% (N = 200)	-	-	-	[64]
	USA, 1999	-	22% (N = 196)	-	-	[55]
**Large text/small text/medical books/video**	USA, 1995	70% (N = 486	17% (486)	-	10% (N = 486)	[52]
	USA, 2009	49% (N = 903)	19% (903)	-	32% (N = 903)	[59]
	USA, 1998	-	16–18% (N = 196)	-	-	[55]
**Social activities (cocktail part, Golf movies)**	USA, 1995	40–41% (N = 486)	42–43% (N = 486)	-	13–15% (N = 486)	[52]
	USA, 2009	4% (N = 903)	68% (N = 903)	-	28% (N = 903)	[59]
	Australia, 2010	15–30% (N = 757)	-	-	-	[61]
	USA, 1998	-	59% (N = 196)	-	-	[55]
**Baby formula**	USA, 1995	41.4% (N = 486)	44.2% (N = 486)	-	10.9% (N = 486)	[52]
**Conference expenses**	USA, 1995	53% (N = 486)	33% (N = 486)	-	12% (N = 486)	[52]
	South Africa, 2011	56% (N = 200)	-	-	-	[64]
	Australia, 2010	75–76% (N = 757)	-	-	-	[61]
	USA, 2009	14% (N = 903)	55% (N = 903)	-	31% (N = 903)	[59]
**Ballpoint pens**	USA, 1995	67.3% (N = 486)	17.5% (N = 486)	-	13% (N = 486)	[52]
	USA, 2009	54.2% (N = 903)	16.2% (N = 903)	-	29.6% (N = 903)	[59]
	Australia, 2010	82% (N = 757)	-	-	-	[61]
	USA, 1998	-	19% (N = 196)	-	-	[55]
**Gift with potential benefit to patient**	Australia, 2010	80–96% (N = 757)	-	-	-	[61]
**Coffee maker**	USA, 1995	39.1% (N = 486)	40.7% (N = 486)	-	17.3% (N = 486)	[52]
**Laptop for home, refrigerator**	Australia, 2010	18–24% (N = 757)	-	-	-	[61]
**Attitude towards the effect of interaction on trust**						
		**Increases trust**	**No effect**	**Decreases trust**	**Neutral**	
	**Country, year**	**% (N)**	**% (N)**	**% (N)**	**% (N)**	**Reference**
**Accepted gifts**	USA, 2008	3–4% (N = 192)	38–48% (N = 192)	47–59% (N = 192)	-	[63]
	Turkey, 2006	-	-	50% (N = 584)	-	[57]
	US, 2006	-	-	Odds ratio: 2.26^3^ (N = 2,029)	-	[62]
**Go on industry-sponsored trips/sporting events**	USA, 2008	3–5% (N = 192)	38–41% (N = 192)	54–58% (N = 192)	-	[63]
**Held stock in drug companies**	USA, 2008	4% (N = 192)	46% (N = 192)	49% (N = 192)	-	[63]
**Industry-sponsored research/lecture**	USA, 2008	7–15% (N = 192)	52–58% (N = 192)	27–40% (N = 192)	-	[63]
**Accepted drug companies meal**	USA, 2008	3% (N = 192)	64% (N = 192)	33% (N = 192)	-	[63]
**Use drug company pen or notepad**	USA, 2008	6% (N = 192)	89% (N = 192)	5% (N = 192)	-	[63]

^1^ This indicates year of the study for each finding (and if not reported, the year of publication)

^2^ N refers to the sample size

^3^ This is the only study in the table that reported the results as Odds ratio rather than percentage

The results of the five studies conducted in the USA are provided as such: fifty-nine (59%) out of 649 members of the general public in 1995 believed that physicians could accept as many office-use gifts as offered while 33% thought the same for personal gifts. On the contrary, 9% and 32% respectively believed that office-use gifts and personal gifts should be limited to less than $25 per year.[54] Forty-three (43%) out of 192 adults sampled from outpatient clinics in 2008 indicated that ‘it was OK for physicians to accept gifts or meals as long as gifts had little monetary value,’ 41% indicated ‘it was not problematic,’ 31% indicated ‘the practice was unethical,’ and 27% indicated that ‘meals made patients wait too long.’[63] That same study also found that participants felt it was “less wrong” for doctors to accept gifts from drug company representatives (mean = 3.7, where higher scores indicate negative judgments) than it was for judges to accept gifts from lawyers, sport referees from players, and politicians from lobbyists (mean = 4.5 for the latter three).[63] Three different studies conducted in 1995, 1998, and 2009 respectively, found that respondents’ approval of gifts were greater than 50% for drug samples, medical books, and pens, and less than 50% for dinners, coffee makers, cocktail parties, golf tournaments, baby formulas and travel.[52, 55, 59] The sample sizes ranged from 196 to 903 respondents.

The two studies conducted in Australia found that respondents were least supportive of gifts that were irrelevant to the medical practice.[58, 61] In the first study conducted in 2007, 26% out of 134 employees of *The Age* newspaper rated free drug samples as being appropriate, while 54.1% rated gifts not relevant to medical practice as being inappropriate. In the same study, 64% to 73% of respondents felt that industry-funded continuing medical education, promotional material provided by pharmaceutical sales representative, and meetings with pharmaceutical sales representative were untrustworthy (scores 1–3 on a 6-point scale, with lower scores indicating untrustworthiness) whereas the majority trusted government funding of such activities.[58] The second study conducted in 2010 compared the attitudes of 832 physicians and 757 members of the general public towards pharmaceutical industry ‘gifts’. While the general public seemed more permissive overall about physicians receiving gifts from the industry, the majority of both physicians and the general public were not supportive of personal ‘gifts’, particularly those that 'were clearly not relevant to medicine’ (e.g. movie tickets to theatre, laptop for home, refrigerator for staff room) even when the costs of these were minimal (e.g. movie tickets).[61]

Three studies were conducted in LMICs. In Pakistan, 88% out of 420 patients attending outpatient settings in 2000 agreed that it was appropriate for doctors to accept gifts from pharmaceutical companies.[56] In Turkey, 71% out of 584 patients admitted to primary health care centers in 2004 agreed that accepting gifts from the drug companies was unethical, yet 83% supported the delivery of free samples given by pharmaceutical companies to people in need.[57] In South Africa, out of 200 postoperative patients surveyed in 2011, the percentages who considered gifts given to physicians to be appropriate differed by type as follows: free attendance at conferences and education classes (56%); free drug samples (46%); small gifts (e.g. pens, notepads) (38%); fees for speaking at industry-sponsored conferences (21%); free food and dinners (12%); and travel or holidays as gifts (2%).[64]

Attitudes towards the effects of interactions on trust (n = 6): The six eligible studies were conducted in the USA (n = 3), Australia (n = 1), Turkey (n = 1) and South Africa (n = 1). Overall, the findings suggest an association between perceived relationship with the industry and decreased trust in physicians (see Table 4).

The three studies conducted in the USA reported the following results: among 192 adults sampled from outpatient clinics in 2008, more than half stated that their trust would decrease if physicians accepted gifts greater than $100 in value or attended industry-sponsored trips and sporting events. Less than half stated that their trust would not change if physicians gave lectures for drug companies, accepted drug companies’ meals, conducted research for drug companies, or used drug companies’ pens or notepads. In addition, 25% said they would be less likely to take a prescribed medication if their physician had recently accepted a gift in return for listening to a pharmaceutical representative’s presentation about that drug.[63] Among 2,029 adults sampled from 10 large metropolitan areas in 2010, those who believed that their personal physicians accepted industry gifts were nearly twice as likely to report low trust in their physicians (OR 2.26, 95% CI 1.56–3.30) and higher distrust in the health care system (OR 2.03 95% CI 1.49–2.77) as compared to those who did not believe that their personal physicians accepted industry gifts.[62] Similar associations were found among participants who believed almost all doctors in general accepted gifts. In the study reporting qualitative data published in 2015, there was a near unanimous agreement among the 31 patients recruited from academic health center’s clinics that physicians who do not voluntarily disclose an existing conflict of interest jeopardize their relationships with patients.[51]

In Australia, 39% out of 134 employees of *The Age* newspaper in 2007 reported that they would choose a doctor who did not see pharmaceutical representatives over one that did. Those who believed information provided by the pharmaceutical industry to be inaccurate were significantly more likely to prefer a physician who did not receive promotional visits.[58]

In Turkey, 50% out of 584 patients admitted to the primary health care centers in 2004 stated they had low confidence in the prescriptions of physicians who accepted gifts from pharmaceutical companies.[57]

In South Africa, 81% out of 200 postoperative patients from four surgical wards in 2011 preferred a physician who had no relationship with, or who did not accept gifts from pharmaceutical companies.[64]

Attitudes towards possible ways to manage the interactions (n = 8): We identified eight studies that assessed the attitudes of patients and the general public towards possible ways to manage physicians’ interactions with the pharmaceutical industry. We categorized the findings under two subheadings: disclosure of interaction (n = 7) and regulation of interactions (n = 1).

Disclosure of interaction: Seven studies assessed participants’ attitudes towards physicians’ disclosure of their interactions with the pharmaceutical industry. These were conducted in the USA (n = 4), Australia (n = 2) and South Africa (n = 1). The majority of participants in four of the seven studies favored physician disclosure of such interactions.

The four studies conducted in the USA reported the following results:

- In a survey of 200 patients admitted to a general medical office in 1995, 74% to 85% thought that physicians participating in post-marketing research should inform patients of the entity paying for the study, whether he or she owns stock, is paid a salary by the sponsoring company, or receives a fee for each patient enrolled, respectively.[53]
- In a survey of 192 adults in the waiting rooms of outpatient clinics in 2008, the percentages of participants who wanted to know whether their physicians accepted gifts varied by type as follows: accepted gifts > $100 in value (51%); attended drug companies social events (46%); went on trips paid by drug companies (43%); accepted gifts less than $100 (36%); gave lecture for drug companies (36%); conducted research for drug companies (35%); accepted drug companies meals (25%); and used drug companies pens or notepads (1%).[63]
- 46% out of 903 adult residents in 2009 answered with a ‘yes’ or ‘maybe’ to the question of whether physicians should disclose personal gifts received from drug companies.[59]
- The majority of the 31 patients in the study reporting qualitative data published in 2015 wanted to know about conflicts of interest that were directly relevant to their care [51].

The two studies conducted in Australia reported the following:

- 84% out of 906 patients in the waiting rooms of three general practices in 2007 felt that it was important for doctors to disclose any relevant competing interests. In addition, 79% wanted to know about any incentives obtained by the doctor. Furthermore, 78% believed that such disclosure would help patients make better informed treatment decisions and 80% stated they would have more confidence in their physicians’ decisions.[60]
- 48% out of 134 employees of *The Age* newspaper in 2007 stated they would prefer to be informed about pharmaceutical marketing.[58]

In the study conducted in South Africa in 2011, 66% out of 200 postoperative patients felt that it was important to know about their physician’s financial relationship with a pharmaceutical company.[64]

Across studies, the most preferred methods of disclosure were ‘simple to read’ printed documents during clinic check-ins [51, 60], and verbally during consultations [51, 60], followed by display on the wall of consulting rooms [60] or in the form of an accredited identification system.[58] In the study with qualitative data published in 2015, patients did not support posting physicians’ conflicts of interest on clinic websites or on signs.[51] They also expressed concerns that verbal disclosure would distract physicians from patient care and/or use up valuable visit time. However, they preferred that physicians bring up the subject of conflict of interest during consultation if such disclosure was directly relevant to a specific aspect of their treatment or care.[51]

Regulation of interaction: We identified one eligible study conducted in Turkey. The study reported that 82% out of 584 patients admitted to primary healthcare centers in 2006 stated that promotional activities aimed at physicians should be forbidden, restricted, or regulated. Stratification of results revealed the following specific percentages: should be forbidden (20%), restricted (25%), or regulated (37%). [57]

### Part 2: Surgeon-device industry interactions

#### Characteristics of included studies

Five studies examined the interactions between surgeons and the device industry. The characteristics of each included study are described in Table 5. All five studies were conducted in North America. One of the studies included patients from both the USA and Canada.[50] Three studies recruited patients from hospitals and clinics while two studies recruited individuals from the general public. All included studies were of quantitative nature and consisted of surveys.[51]

**Table 5:** Characteristics of included studies on surgeons’ interactions with the device industry.

Study ID/	Sample size and funding	Participants and settings	Sampling frame and method	Type of interaction	Outcomes assessed
**Khan, 2007[47]**	• N = 245 • Funding not reported	• Patients in the waiting area in orthopedic surgery clinic • USA • 51.0% female; average age: 55.5 (±14.5) • Education: 33.9% collegegraduates; 19.2% graduate/postgraduates	• Frame: Patients in the waiting area in orthopedic surgery clinic • Method: Convenient sampling	Surgeons as consultants for industry and medical device manufacturers	• Attitudes towards the interactions • Attitudes towards possible ways to manage the interactions - Regulation of interaction - Entities that should be involved in regulating interaction
**Fisher, 2012[49]**	• N = 501 • Funding not reported	• North American public visiting the spineuniverse.com website • USA; 2 weeks (date not reported) • 63.3% females; 46.9% aged 30–49, 26.1% aged 50–59, and 19.2% aged 60 and above; • Education: 52% tech or 4-year college; 25.7% graduate	• Frame: North American public visiting the spineuniverse.com website. • Method: convenient sampling	Surgeon-industry COI relating to the role of surgeons in clinical research and the industry funding of such research	• Beliefs about their effects on quality of care • Attitudes towards the interactions • Attitudes towards possible ways to manage the interactions - Regulation of interaction - Entities that should be involved in interaction
**Camp, 2013[50]**	• N = 251 USA and N = 252 Canada • No external funding sources	• Postoperative arthroplasty patients attending follow up hip and knee arthroplasty clinics • USA & Canada; Nov 2010-March 2011 • 55% females US; 59% females Canada; age: 36% < 60, 64% 60 and above for US; 30% < 60, 69% 60 and above for Canada • Education: US (51% college /university degree; Canada (51% college/university degree	• Frame: postoperative patients attending follow up hip and knee arthroplasty clinics at Mount Sinai Hospital and Holland Orthopaedic • Method: Convenient sampling	Financial relationships with manufacturers (gifts, royalties, consultancy payments, speakers’ bureau presentations, or research support)	• Awareness of the interactions of surgeons in general • Beliefs about their effects on quality of care • Attitudes towards the interactions • Attitudes towards possible ways to manage the interactions - Disclosure of interaction - Regulation of interaction - Entities that should be involved in regulation the interaction
**Lieberman, 2013[48]**	• N = 100 • Funding from the NIH Musculoskeletal Transplant Foundation	• Patients (18 years old or older) scheduled for primary THA and TKA from the orthopedic practices of two joint arthroplasty specialists • USA; September 2010 to September 2011 • 66% female; mean age (SD): 63 (±13.3) • Education: 49% college; 20% Master’s or Doctoral degree	• Frame: All patients 18 years and older scheduled for primary THA and TKA from orthopedic practices of two joint arthroplasty specialists at an academic health center. • Method: convenient sampling	• Developed prostheses • Receives revenue from company • Stock in company • Receive future revenue • Paid for product used in surgery • Paid for product not used in surgery	• Awareness of the interactions of surgeons in general • Attitudes towards the interactions • Attitudes towards the effects of interactions on trust • Attitudes towards possible ways to manage the interactions - Disclosure of interaction
**Dipaola, 2014[7]**	• N = 610 • Funding not reported	• North Americans representing the general public visiting the spineuniverse.com website • USA; 2 weeks (no data) • 63.3% females; 42.8% aged 30–49, 31% aged 50–59, 21% aged> 60 • Education: 54.8% technical school college; 24.6% graduate school	• Frame: visitors of the spineuniverse.com website. • Method: convenient sampling	Surgeon-industry consulting relationships	• Beliefs about their effects on quality of care • Attitudes towards the interactions • Attitudes towards possible ways to manage the interactions: - Disclosure of interaction - Entities that should be involved in regulating interaction

The included studies assessed the following types of outcomes: knowledge (n = 2), beliefs (n = 3), and attitudes (n = 5) of patients and the general public regarding the interactions between surgeons and the device industry. Five studies also examined the attitude of patients and the general public towards possible ways to manage these interactions (e.g., disclosure and regulation of surgeon-industry interactions). All of the studies assessed more than one type of outcome.

#### Methodological features

The methodological features of the five included studies are described in S3 Table.

While all five studies examining the relationship with the device industry described their sampling frame, fewer fulfilled the following factors related to the risk of bias: reporting using a partially validated tool (n = 2); [7, 49] and reporting pilot testing the tool (n = 3). [7, 49, 50] None of the studies reported sample size calculation or using a random approach to sampling. The response rates for two of the studies were 41% [48] and 90% [50], respectively. The remaining three studies did not report their response rates (two of which included participants visiting the spineuniverse.com website).

#### Summary of findings

We provide a detailed summary of the findings for each included study in S4 Table. We also provide below a narrative synthesis of the results organized according to the outcomes of interest described under the eligibility criteria section above:

- Awareness of the interactions between surgeons and the device industry (n = 2)
- Beliefs about the effects of interactions on quality of care (n = 3)
- Attitudes towards the interactions (n = 5)
- Attitudes towards the effects of interactions on trust (n = 1)
- Attitudes towards possible ways to manage the interactions (n = 5)

Awareness of the interactions between surgeons and the device industry (n = 2): The two eligible studies were conducted in North America between 2010 and 2011. In the first study, 47% out of 100 patients from orthopedic practices of two joint arthroplasty specialists were knowledgeable of a financial conflict of interests related to clinical research of surgical device manufacturers. In addition, 13% stated they had received information from a surgeon regarding financial conflict of interests in relation to research for a surgical device in the past.[48] In the second study, 54% out of 251 patients admitted to post-operative clinics in the USA and 36% out of 252 in Canada were aware of surgeons’ interactions with device manufacturers.[50]

Beliefs about the effects of interactions on quality of care (n = 3): The majority of participants in the three studies believed that their care would either improve or not be affected if surgeons interacted with the device industry (see Table 6).[7, 49, 50] The findings of each study are presented below:

- 76% out of 251 patients in the USA and 74% out of 252 in Canada admitted to post-operative clinics between 2010 and 2011 felt that their surgeon would make the best choices for their health, regardless of financial relationships with device manufacturers.[50]
- 55% out of 501 participants visiting the spineuniverse.com website in 2012 believed that the surgeon’s source of medical research funding would affect their quality of care, with 82% stating that medical industry-funded research could be valuable for patients.[49]
- In a survey of 610 participants visiting the spineuniverse.com website in 2014, 61% to 80% believed that their care would either improve or not be affected if their surgeon was a consultant to help design/improve a surgical device (80%), if royalties were paid to the surgeon when he/she used the product (61%), or if surgeons received royalties on devices that they designed but were implanted by other surgeons (75.4%).[7]

**Table 6:** Beliefs of patients and the general public about the effects of surgeon-device industry interactions on quality and cost of care.

**Beliefs about the effects of surgeon-device industry interaction on quality of care**						
**Type of interaction**		**Positive effect**	**No effect**	**Negative effect**	**Don’t know**	
	**Country, year**^**1**^	**% (N)**^**2**^	**% (N)**	**% (N)**	**% (N)**	**Reference**
**Surgeon as consultant to help design /improve a surgical device**	USA, 2014	46.4% (N = 610)	34.1% (N = 610)	19.5% (N = 610)	-	[7]
**Royalties are paid to surgeon when he/she uses the product or other surgeons use the product**	USA, 2014	17.7–17.9% (N = 610)	43.1–57.5% (N = 610)	24.6–39.2% (N = 610)	-	[7]
		**Influences decision/care**	**May influence decision/care**	**Has little or no influence**	**Not sure/No opinion**	
	**Country, year**	**% (N)**	**% (N)**	**% (N)**	**% (N)**	**Reference**
**Financial relationships with device manufacturers**	Canada, 2010–2011	-	-	74% (N = 252)	-	[50]
	USA, 2010–2011	-	-	76% (N = 251)	-	[50]
**Source of medical research funding for a study**	USA, 2012	55% (N = 501)	-	30.5% (N = 501)	14.4% (N = 501)	[49]

^1^ This indicates year of the study for each finding (and if not reported, the year of publication)

^2^ N refers to the sample size

Attitudes towards the interactions (n = 5): The five studies were conducted in North America. The majority of respondents in these studies were not concerned about financial conflicts of interest between their surgeons and device manufacturers (see Table 7). [7, 47–50] In the first study, 94% out of 245 patients in the waiting area of an orthopedic clinic in 2007 thought it was beneficial for them if doctors advised the medical device manufacturers to improve/design medical instrumentation and 67% believed that physicians should be compensated for this advisory role.[47] In the second study, 51% out of 100 patients sampled from orthopedic practices between 2010 and 2011 preferred to be operated on by a surgeon who had developed prosthesis whereas 40% and 43% would be concerned if respectively, a surgeon had a financial relationship with a company or was paid by a company that manufactured a product used in the surgery.[48] In the third study, 76% out of 251 post-operative patients attending follow-up clinics between 2010 and 2011 in the USA and 74% out of 252 in Canada felt their surgeon would make the best choices for their health, regardless of financial relationships with device manufacturers.[50] The remaining two studies included individuals from the general public visiting spineuniverse.com website; 91% out of 501 individuals in 2012 felt that surgeons’ input was important for industry-funded research [49]; 82% out of 610 individuals in 2014 felt that it was ethical for surgeons to work with companies as consultants to design or improve health-care products or devices.[7]

**Table 7:** Attitudes of patients and the general public towards surgeons’ interactions with the device industry.

**Attitude towards the appropriateness/acceptability of surgeon-device industry interaction**						
**Type of interaction**		**Appropriate/Acceptable**	**Not appropriate/Not acceptable**	**No answer**	**Unsure**	
	**Country, year**^**1**^	**% (N)**^**2**^	**% (N)**	**% (N)**	**% (N)**	**Reference**
**Work/advise/provide input to the medical device manufacturers to improve/design medical instrumentation**	USA, 2007	94.3% (N = 245)	-	-	5.7% (N = 245)	[47]
	USA, 2012	90.6% (N = 501)	3% (N = 501)	6.4% (N = 501)	-	[49]
	USA, 2014	81.8% (N = 610)	18.2% (N = 610)	-	-	[7]
**Receive compensation from the company for advisory role**	USA, 2010–2011	48% (N = 251)	32% (N = 251)	21% (N = 251)	-	[50]
	Canada, 2010–2011	53% (N = 252)	25% (N = 252)	22% (N = 252)	-	[50]
	USA, 2007	66.5% (N = 245)	9.4% (N = 245)	0.8% (N = 245)	23.3% (N = 245)	[47]
**Be allowed to recommend the use of a device he/she helped to design**	USA, 2007	89.4% (N = 245)	1.2% (N = 245)	9% (N = 245)	0.4% (N = 245)	[47]
**Own shares in the company that madeyour hip or knee replacement**	USA, 2010–2011	21% (N = 251)	49% (N = 251)	30% (N = 251)	-	[50]
	Canada, 2010–2011	22% (N = 252)	48% (N = 252)	30% (N = 252)	-	[50]
**Get payments from the company to give lectures, including some that might discuss the company’s products.**	USA, 2010–2011	46% (N = 251)	31% (N = 251)	23% (N = 251)	-	[50]
	Canada, 2010–2011	53% (N = 252)	26% (N = 252)	21% (N = 252)	-	[50]
**Get payments from the company for a patent on a product that your surgeon designed**	USA, 2010–2011	69% (N = 251)	16% (N = 251)	15% (N = 251)	-	[50]
	Canada, 2010–2011	66% (N = 252)	15% (N = 252)	19% (N = 252)	-	[50]
**Get gifts worth more than $100 from the company that made your hip or knee replacement.**	USA, 2010–2011	11% (N = 251)	63% (N = 251)	26% (N = 251)	-	[50]
	Canada, 2010–2011	13% (N = 252)	59% (N = 252)	28% (N = 252)	-	[50]
**Get gifts worth less than $100 from the company that made your hip or knee replacement**	USA, 2010–2011	20% (N = 251)	51% (N = 251)	29% (N = 251)	-	[50]
	Canada, 2010–2011	18% (N = 252)	46% (N = 252)	35% (N = 252)	-	[50]
**Be allowed to perform research on products in which they have a financial interest without regulations**	USA, 2012	20% (N = 501)	66.1% (N = 501)	14% (N = 501)	-	[49]
		**Less likely to have a surgeon operate on them**	**Neutral**	**More likely to have a surgeon operate on them**		
	**Country, year**	**% (N)**	**% (N)**	**% (N)**	**% (N)**	**Reference**
**Surgeon developed prostheses**	USA, 2010–2011	14% (N = 100)	34% (N = 100)	51% (N = 100)	-	[48]
**Surgeon received: revenue from company; payment for product used or not used in surgery; or has stock in company**	USA, 2010–2011	25–44% (N = 100)	31–45% (N = 100)	19–30% (N = 100)	-	[48]

^1^ This indicates year of the study for each finding (and if not reported, the year of publication)

^2^ N refers to the sample size

Attitudes towards the effects of interactions on trust (n = 1): We identified one eligible study conducted in the USA. The study reported that 24% out of 100 patients sampled from orthopedic practices between 2010 and 2011 indicated they would trust a surgeon less if he or she had financial conflicts of interest (44% disagreed). Also, 15% of the patients believed that such conflicts would make them less likely to have their surgeon operate on them.[48]

Attitudes towards possible ways to manage the interactions (n = 5): We categorized the findings under three subheadings: disclosure of interaction (n = 3), regulation of interactions (n = 3), and entities that should be involved in regulation of interactions (n = 4).

Disclosure of interaction: The majority of participants in two of the three studies supported disclosure of surgeons’ interaction with the device industry. The findings of each study are presented below [7, 48, 50]:

- 47% out of 251 patients attending follow-up arthroplasty clinics between 2010 and 2011 in the USA and 42% out of 252 in Canada wanted surgeons to verbally disclose financial relationships with manufacturers.[50] However, only 33% and 32% respectively ‘agreed’ or ‘strongly agreed’ that if their surgeon’s financial relationships were on a public web, they would look at the web site before deciding to go ahead (36% and 43% of respectively ‘disagreed’ or ‘strongly disagreed’).
- 55% out of 100 patients sampled from orthopedic practices between 2010 and 2011 believed surgeons should make patients aware of financial conflicts of interest.[48]
- 62% out of 610 participants visiting the spineuniverse.com website in 2014 felt surgeons should disclose consulting relationships to all patients regardless of whether they plan to use the devices in their own surgery.[7]

Only one of the studies assessed participants’ preferred methods of disclosure.[50] Forty-seven (47%) out of 251 postoperative patients sampled between 2010 and 2011 in the USA and 42% out of 252 in Canada wanted their surgeon to verbally disclose financial relationships with manufacturers, whereas 42% and 38% respectively wanted this disclosure in the form of a pamphlet.

Regulation of interactions: Three studies conducted in North America assessed participants’ attitudes towards the regulation of surgeons’ interactions with the device industry. In the first study conducted in 2007, 19% out of 245 patients in the waiting area of an orthopedic surgery clinic believed that physician interactions with medical device manufacturers should not be subject to any regulations while 48.2% thought they should be regulated and 32% were unsure.[47] In the second study, 20% out of 501 participants visiting the spineuniverse.com website in 2012 felt that surgeons should be allowed to perform research on products in which they have a financial interest without limitations by outside regulatory guidelines or agencies.[49] The latter percentage rose to 69% as long as “guidelines are set up to regulate potential conflict of interest”. In the third study, 38% out of 251 postoperative patients attending follow up clinics between 2010 and 2011 in the USA and 30% out of 252 in Canada agreed that surgeons should place their financial relationships on a publicly accessible web site.[50]

Entities that should be involved in regulating the interaction: The four studies were conducted in North America.[7, 47, 49, 50] The majority of participants in the four studies favored the involvement of professional rather than governmental bodies in regulating the interactions between surgeons and the industry (see Table 8).

**Table 8:** Attitudes of patient and the general public towards the entities that should be involved in regulating surgeon-device industry interactions.

**% of patients who agreed that the following entities should be involved in regulation of interaction**								
	**Physicians**	**Professional organization**	**Hospitals or universities**	**Government**	**Company representative**	**No answer**	**Combination of the latter**	
**Country, year**^**1**^	**% (N)**^**2**^	**% (N)**	**% (N)**	**% (N)**	**% (N)**	**% (N)**	**% (N)**	**Reference**
USA, 2010–2011	81% (N = 252)	83% (N = 251)	60% (N = 251)	26% (N = 251)	-	-	-	[50]
Canada, 2010–2011	78% (N = 252)	83% (N = 252)	61% (N = 252)	35% (N = 252)	-	-	-	[50]
USA, 2007	32.2% (N = 245)	-	20% (N = 245)	13.5% (N = 245)	-	34.3% (N = 245)	-	[47]
USA, 2012	-	11.6% (N = 501)	6% (N = 501)	8.2% (N = 501)	2.6% (N = 501)	-	71.7% (N = 501)	[49]
USA, 2014	-	17.4% (N = 610)	7.4% (N = 610)	10% (N = 610)	1% (N = 610)	-	64.3% (N = 610)	[7]
**% of patients who agreed that the following entities should have the most power to regulate interaction**								
		**Medical professional societies**	**Hospitals or universities**	**Government**	**Company representative**	**Not sure**		
**Country, year**	**% (N)**	**% (N)**	**% (N)**	**% (N)**	**% (N)**	**% (N)**	**% (N)**	**Reference**
USA, 2014	-	34.9% (N = 610)	13% (N = 610)	17.9% (N = 610)	1.5% (N = 610)	32.7% (N = 610)	-	[7]
USA, 2012	-	34.1% (N = 501)	13.1% (N = 501)	8.7% (N = 501)	0.8% (N = 501)	43.3% (N = 501)	-	[49]

^1^ This indicates year of the study for each finding (and if not reported, the year of publication)

^2^ N refers to the sample size

Two of the four studies included participants visiting SpineUniverse website in 2012 (N = 501) and 2014 (N = 392) and used similar questions. 64% to 71% of participants in 2012 and 2014 respectively thought that a combination of groups including doctors, universities, government, and company representatives should be involved in regulating financial conflicts of interest.[7, 49] The top selected single entity that should have the most regulatory power was medical professional societies. The top selected entities that ‘should not be involved’ were medical company representatives followed by government representatives.[7, 49] The percentages of participants who believed that medical company representatives should not be involved in regulating surgeon-industry relationships rose from 30% in 2012 to 45% in 2014.[7, 49] In the third study conducted in 2007, the percentages out of 245 patients attending orthopedic clinics who thought the following entities should be involved in regulating physician-medical device industry relationship were: physicians (32%); hospitals (20%); government (13.5%); and no answer (34%).[47] In the fourth study, 83% out of 251 postoperative patients attending follow-up clinics between 2010 and 2011 in the USA and and 83% out of 252 in Canada wanted their surgeon’s professional organization to ensure that financial relationships were appropriate, while 26% and 35% respectively favored monitoring by a government agency.[50]

## Discussion

### Summary and interpretation of findings

Our systematic review identified twenty studies assessing the knowledge, beliefs and attitudes of patients and the general public towards physicians’ interactions with the pharmaceutical and the device industry. Many of these studies failed to meet methodological safeguards for protecting from bias thus the findings should be interpreted with caution.

Interactions with the pharmaceutical industry: the percentages of participants reporting being aware of physicians’ interactions with the industry were lower for *own* physician compared to physicians in general. Also, higher percentages of participants reported awareness of educational and office-use gifts relative to personal gifts. Although not a consistent finding across studies, participants were more likely to believe that physicians’ interactions with the pharmaceutical industry increased cost of care, with mixed findings for their effects on prescribing behaviors and quality of care.

The review also found that participants were more accepting of office-use gifts and gifts of potential benefits to patients relative to personal gifts. While the public may not necessarily perceive the influence of small and office-use gifts as negative, the social science literature suggests that even gifts of little value can influence physicians’ behaviors in the spirit of reciprocity. [46, 66] We also found a potential association between perceived relationships with the industry and lower trust in physicians. Generally, participants supported physicians’ disclosure of their interactions with the pharmaceutical industry, preferably through easy-to-read printed documents and verbally during consultation visits.

Interactions with the device industry: the percentages of participants reporting being aware of surgeon’s interactions with the device industry ranged from 35% to 57%. The majority of patients felt their surgeons would make the best choices for their health, regardless of their financial relationships with the device industry. While this may reflect trust in the surgeon or satisfaction with the clinical outcome, according to one study, it might be the result of misinformation or lack of information conveyed to patients. [48] Generally, participants supported physicians’ disclosure of their interactions with the device industry, and preferred professional rather than governmental bodies to regulate surgeon-industry conflict of interest.

### Comparisons of the two industries

We identified only one study assessing differences of awareness by type of industry. Camp et al surveyed patients who underwent surgery about their views on surgeons’ interactions with both the pharmaceutical and the device industry.[50] Fewer participants reported being aware of surgeons’ financial relationships with device companies than being aware of doctors’ financial relationships with drug companies. The investigators attributed such differences to the higher media coverage for the interactions with the pharmaceutical industry.

We attempted to compare studies that assessed the interactions with the pharmaceutical industry with studies that assessed the interactions with the device industry. Acknowledging that any differences in findings could be due to differences in populations, countries, year, and method used, there appears to be less concern and more acceptance of surgeons’ interactions with the medical device industry compared to physicians’ interactions with the pharmaceutical industry.

There might be different explanations for this observation. First, it is likely that individuals do not fully comprehend the potential conflict of interest and biases that may occur between surgeons and the device industry and the implications of such interactions on their clinical care.[48] Similarly, patients may perceive a surgeon’s participation in surgical device development as an indication of a higher level of clinical expertise, or a better understanding of its use.[48] On the other hand, many patients facing the immediate prospect of a surgical procedure may view the issue of conflict of interest as an unnecessary and unwanted burden which shifts the focus away from more important priorities.[51, 67]

### Time and geographical trends

While we originally planned to explore trends over time and by country, ideally, we would need studies using the same instrument in the same population over time (for temporal trends) or in different countries at the same time (for geographical trends). We did find two studies conducted in the United States 15 years apart. [52, 59] Although the two studies did not include the same population, they used similar questions. The findings suggest improved awareness over time of the different types of gifts. Also, we identified two studies conducted in North America in 2012 and 2014, respectively, that included participants visiting SpineUniverse website and used similar questions. The percentage of participants that believed that medical company representatives should not be involved in regulating surgeon-industry relationships rose from 30% in 2012 to 45% in 2014. [7, 49]

In terms of geographical trends, we identified one study that assessed the awareness and attitude of patients in both Canada and the United States using the same survey tool.[50] The investigators found a higher level of awareness among US patients as compared to Canadian patients of financial relationships with the pharmaceutical industry (70% versus 55% respectively) and the device industry (54% versus 35% respectively).

### Influence of culture

We acknowledge that knowledge, beliefs and attitudes are inherently influenced by culture. While we did not identify any paper addressing the effect of culture on the knowledge, beliefs and attitudes of patients and the general public, the included surveys may provide some clues. Sixty-two percent (62%) of respondents to a survey conducted in Pakistan, agreed that a “doctor is next to God.”[56] Consistently, 88% agreed it is appropriate for doctors to accept gifts from pharmaceutical companies. On the contrary, 71% of patients responding to a survey in Turkey agreed that accepting gifts from drug companies was not ethical.[57]

### Comparison to findings of similar reviews

We identified two previously published systematic reviews related to our topic. While our review focused specifically on practicing physicians, the other two included medical researchers.[39, 43] The searches for those reviews date back to 2007 and 2009 respectively. Our review has included ten studies published since that date.[7, 48–51, 61–65] Our search also identified three studies [47, 56, 58] published prior to 2009 but not included in those reviews. Finally, one of these reviews did not assess the methodological features of the included studies.[43] In terms of findings, our systematic review confirmed previous findings while contributing new information. The results of the sixteen studies focusing on interactions with the pharmaceutical industry were consistent with those of the two previously published systematic reviews in terms of greater acceptability and fewer perceived influence for smaller, less costly office-use gifts compared to personal gifts. [39, 43] Another consistent finding was the heterogeneity in the perceived effects of physician- industry interactions on prescription behaviors, quality of care and cost of care. One other common finding was the general desire for disclosure of such interactions among participants.

One area not covered by the findings of the two previous reviews was the interactions between surgeons and the device industry. Additional new information relates to participants’ preferred methods of disclosure of physician-industry interactions in clinical care, and the entities that should be involved in regulating physician-industry interactions. Finally, our findings point to a potential association between perceived relationships with pharmaceutical industry and decreased trust in physicians, which warrants further attention due to its potential influence on patients’ clinical decisions.

### Strengths and limitations

The major strength of this systematic review is the use of standard Cochrane Collaboration methodology, including a risk of bias assessment of included studies. One limitation of our review is the exclusion of studies published in languages other than English. Another limitation relates to shortcomings of the included primary studies, e.g. low response rates, the use of convenience samples, and the use of non-validated tools. Also, the majority of studies were from high-income countries which may affect the generalizability of findings to low and middle and income settings. Finally, the heterogeneity of the populations, contexts, and measurement tools precluded sufficient comparisons of findings across studies and over time.

### Implications for policy and practice

The findings have implications at the patient, provider, institutional and health system levels. At the patient level, there should be more efforts to increase awareness and education about the potential benefits and more importantly, adverse effects of physician-industry interactions on prescribing quality and cost of care to allow for more informed decision-making. This might be especially relevant to interactions between surgeons and the device industry. For the latter, it is important for patients to understand and distinguish between interactions that benefit current or future patients and those that benefit the surgeon or device manufacturer and promote unethical behavior. While we could not identify interventions specifically targeting this aspect, studies to improve patient awareness and attitudes towards generic drugs found that media campaigns and educational sessions led to the increased use of generic drugs.[68, 69]

At the provider level, there is also a need to raise awareness among physicians and other healthcare professionals on how their interactions with the industry may negatively influence their behavior [18, 65] and their relationships with patients.[48, 62] Existing evidence shows positive effects of educational programs about industry marketing strategies (e.g., seminars, role playing, and focused curricula) on physician trainees’ attitudes and behaviors.[70–72] Another approach would be for providers to verbally disclose to patients their interactions with the industry through ‘easy to read’ printed materials during clinic check-ins or verbally during consultations. [50, 51, 60]

At the institutional level, existing evidence suggests a positive effect of restrictive institutional policies governing physician-industry interactions on physician prescription behavior [38] and medical students' support for banning contacts with pharmaceutical representatives.[72] Considerations could also be given to disclose the interactions between physicians and the industry as part of the ethical framework of health care organizations, which is increasingly being promoted by healthcare accreditation programs.[21, 67, 73, 74] On a similar note, professional organizations could assume a stronger role in regulating surgeon-industry interactions as revealed by our review whereby the majority of participants, albeit from North America, supported such a role.

Finally, potential system level interventions include self-regulation (e.g., voluntary codes of practice), and governmental regulations (e.g., outright ban on physician gifts, required disclosure of physician-industry interactions, limits on the sale of prescribing data for marketing purposes and public funding of academic detailing programs).[75] However, evidence shows that industry self-regulation and voluntary guidelines for sales representatives may not always be sufficient to properly regulate physician-industry interactions.[37, 46, 70, 76, 77] Having said this, there are ongoing experiences that are worth monitoring and studying, e.g., the Australian manufacturers’ Code of Conduct.[37]

One governmental approach worth noting is the Sunshine Act enacted in 2010 in the USA. This was the first Congressional involvement in regulating the disclosure of payments exceeding $10 per instance or $100 per year by pharmaceutical and device companies to physicians and teaching hospitals. However, the impact of the Sunshine Act is yet to be assessed.

### Implications for research

Given that the majority of the included studies were conducted in high-income countries, future studies should explore the knowledge, beliefs and attitudes of patients and the general public towards physician-industry interactions in low- and middle-income countries. In addition, there is a need to improve the quality of studies in this field, particularly in terms of using validated survey tools.

More research is needed to explore the patients’ and the general public’s level of understanding of the interactions between surgeons and the device industry which remains a relatively understudied area. Future research could explore the most preferred format to disclose physician-industry interactions to patients and the general public as well as the impact of such disclosures on patients’ decision-making regarding their clinical care. Finally, it is important to explore the influence of culture on the knowledge, beliefs and attitudes of patients and the general public. An interesting distinction could be made between traditional and non-traditional cultures where the former tend to display more respect for authority.[78]

## Supporting Information

S1 Appendix Search strategies. (PDF)

S2 Appendix Excluded studies and reasons for exclusion. (PDF)

S3 Appendix PRISMA checklist. (PDF)

S1 Table Methodological features of included studies on interactions with the pharmaceutical industry. (PDF)

S2 Table Summary of findings on interactions with pharmaceutical industry. (PDF)

S3 Table Methodological features of included studies on interactions with device industry. (PDF)

S4 Table Summary of findings on interactions with device industry. (PDF)
