# Supplementary material for: Knowledge, Beliefs and Attitudes of Patients and the General Public towards the Interactions of Physicians with the Pharmaceutical and the Device Industry: A Systematic Review
Source: PLoS One. 2016 Aug 24;11(8):e0160540. doi: 10.1371/journal.pone.0160540 (PMC4996522; doi:10.1371/journal.pone.0160540)
Supplement: S1 Appendix — (PDF) [file pone.0160540.s001.pdf]

S1 Appendix: Search strategies

Ovid MEDLINE(R) <1946 to August Week 4 2015>

1. Conflict of Interest.mp. or “Conflict of Interest”/ (9234)
2. Drug Industry/ (30016)
3. Gift Giving/ (1306)
4. detailman.mp. (4)
5. commercial information.mp. (30)
6. ((drug or pharma*) adj3 (industry or firm* or manufacture* or compan*)).mp. (40649)
7. physician*.mp. (423538)
8. doctor*.mp. (89855)
9. Physicians/ (65190)
10. primary care.mp. (72237)
11. or/1-6 (48354)
12. or/7-10 (522090)
13. 11 and 12 (5684)
14. 13 not (comment or editorial or letter).pt. (4745)

Embase <1980 to 2015 Week 36>

1. Conflict of Interest.mp. or “Conflict of Interest”/ (9927)
2. Drug Industry/ (68082)
3. Gift Giving/ (962)
4. detailman.mp. (3)
5. commercial information.mp. (47)
6. ((drug or pharma*) adj3 (industry or firm* or manufacture* or compan*)).mp. [mp=title, abstract, heading word, drug trade name, original title, device manufacturer, drug manufacturer, device trade name, keyword] (97838)
7. physician*.mp. (519656)
8. doctor*.mp. (200550)
9. Physicians/ (163052)
10. primary care.mp. (102606)
11. or/1-6 (106455)
12. or/7-10 (708268)
13. 11 and 12 (8296)
14. 13 not (comment or editorial or letter).pt. (7157)
