# Supplementary material for: Knowledge, Beliefs and Attitudes of Patients and the General Public towards the Interactions of Physicians with the Pharmaceutical and the Device Industry: A Systematic Review
Source: PLoS One. 2016 Aug 24;11(8):e0160540. doi: 10.1371/journal.pone.0160540 (PMC4996522; doi:10.1371/journal.pone.0160540)
Supplement: S2 Appendix — (PDF) [file pone.0160540.s002.pdf]

S2 Appendix. Excluded studies and reasons for exclusion

Reasons for exclusion

1= No outcome of interest (n=25)

2= Not population of interest (n=54)

3= Systematic review (n=1)

4= Association study (n=32)

5= Not in English (n=5)

**Name**	**Year**	**Exclusion number**	**Detailed reason for exclusion**
Aasland [1]	2004	5	Not in English
Al-Areefi [2]	2013	2	Beliefs and attitudes of physicians
Alghasham [3]	2009	1	Potential pressures from patients and drug companies on physician generic drug prescription
Alssageer,[4]	2013	2	Beliefs of physicians
Al Zaharani[5]	2014	4	Impact of pharmaceutical industry on physician prescribing behaviors
Andaleeb[6]	1995	2	Attitudes of physicians towards pharma sale representatives
Anderson[7]	2009	2	Attitudes and beliefs of physicians regarding factors associated with self-reported reliance on representatives when making prescribing decisions
Arkinson[8]	2010	3	Systematic review
Backer [9]	2000	2	Attitude of physicians
Banks[10]	1992	2	Attitudes of physicians towards gift from pharma
Berlings[11]	1994	4	Effect of industry-independent drug information on physicians' prescription of drugs
Brett[12]	2003	2	Attitudes and beliefs of physicians towards gifts from pharma
Burashnikova[13]	2008	2	Attitudes of physicians and residents towards pharmaceutical promotion techniques
Buusman[14]	2007	1	How GPs choose between drugs

Caamano [15]	2002	4	Influence of commercial information on prescription quantity
Campo [16]	2006	4	Physicians’ decision process for drug prescription and impact of pharma marketing mix instruments
Carroll[17]	2007	4	Impact of educational interventions on medical trainees’ attitudes and behaviors regarding industry-trainee and industry-physician relationships
Carthy[18]	2000	1	Modifiable factors associated with GP prescribing variance and cost
Castresana [19]	2005	5	Spanish
Caudill [20]	1996	2	Attitude of physicians
Chimonas[21]	2007	1	Physicians’ techniques for managing cognitive inconsistencies within their relationships with drug representatives
Chren[22]	1994	4	Physicians' behavior and their interactions with drug companies
Civaner[23]	2012	1	Sales strategies of pharmaceutical companies
Creyer EH[24]	1998	4	Sales representatives' influence on physicians' impressions of the industry
De Las Cuevas [25]	2002	4	Pressure of promotions by the pharmaceutical industry
De Gara [26]	2013	2	Physician perceptions surrounding conflict of interest.
Ellison[27]	2009	4	Rates of reporting commercial bias by physicians following online CME activities
Evans[28]	2011	1	Benefits of refusing visits from pharma
Evans[29]	1987	4	Influence of prescription drug information source on the usefulness of the information
Fagundes[30]	2006	1	Bioethical analysis of drugs advertisement and publicity
Fassold[31]	1996	2	Attitudes of physicians towards drug promotion
Fernandez,[32]	2000	1	Opinion of physicians regarding the importance and legitimacy of sources of influence on medical practice
Figueiras, [33]	2000	4	Influence of physician’s education, drug info and medical-care setting of the quality of drugs prescribed
Fischer[34]	2009	2	Attitudes and beliefs of physicians
Fortuna[35]	2007	4	Influences on prescribing decisions
Freeman[36]	1993	2	Opinion of physicians regarding the importance of several items on their prescribing of drug for patients with panic disorder

Fugh-Burman[37]	2011	4	Promotional tone in reviews of menopausal hormone therapy
Gaedeke[38]	1999	1	The perceived value of specific services provided by pharma sales rep
Gaither[39]	1994	2	Beliefs and attitudes of physicians
Gaither[40]	1996	2	Attitude of physicians
Goluksuz[41]	2009	5	Not in English
Gray[42]	2007	2	Evaluates how advanced cancer patients enrolled onto early-phase clinical trials of experimental agents perceive investigator COI
Guldal[43]	2000	4	Influences of drug companies ads programs on physicians
Gundermann[44]	2010	5	Not in English
Gunnardottir[45]	2005	1	Physicians' perception of the influence of several variables on their prescribing behavior
Haayer[46]	1982	4	Rational prescribing and sources of information
Hajjaj[47]	2010	1	Nonclinical influences in dermatology decision making
Hall[48]	2006	2	Perceptions of the influence of prescription medicine sample on prescribing
Halperin[49]	2004	2	Opinion of physicians towards gift giving
Hampson, [50]	2006	2	Examine views of patients in research trials regarding potential conflicts of interest between researchers or medical centers and drug companies
Hassali[51]	2006	1	Factors influencing prescribing decisions
Hayes[52]	1990	2	Attitude of physicians towards role of the pharma industry in CME
Herrera[53]	2004	5	Not in English
Hodges[54]	1995	2	Attitude of physicians towards pharma-physician interactions
Hull[55]	1987	1	Sources of information about new drugs and physician attitudes towards drug prescribing
Jones[56]	2001	1	Physicians’ perceptions of the factors that influence their decisions to introduce new drugs into their clinical practice

Jones[57]	2000	4	Factors influencing prescribing decisions: new drugs
Joubert[58]	1984	2	Attitudes of physicians
Kao[59]	2011	2	Attitudes and beliefs of residents toward pharma marketing practices
Katz[60]	2002	2	Attitudes of physicians towards academia-industry: collaboration in CME
Keim[61]	2004	2	Attitudes and beliefs of physicians
Kelly[62]	1996	1	Motivation of physicians attending education events
Kelmenc-Ketis, Z., [63]	2013	4	Influence of interaction on physicians' prescribing patterns
Kerridge[64]	2005	1	Nature and extent of relationships between pharmaceutical industry and medical organizations
Kersnik[65]	2011	2	Family doctors' views of pharmaceutical industry sales representatives
Keune[66]	2011	2	Attitudes and beliefs of physicians towards disclosing financial conflicts of interest
Kim,[67]	2004	2	Research participants
Kish-doto[68]	2008	1	Investigation of four dimensions of psychiatrists' prescribing practices for antiepileptic drugs
Klein[69]	2006	4	Factors influencing prescribing decisions
Korenstein[70]	2010	2	Attitudes of physicians towards the pharmaceutical industry
Kyle[71]	2008	4	Influence of pharmaceutical industry on drug prescription
Kyle[72]	2009	4	Influence of marketing expenditure on drug prescription
Lexchin[73]	1997	1	Information received by physicians from pharmaceutical industry
Loh[74]	2007	4	Impact of CME activities on clinical practice
Lurie[75]	1990	4	Nature, frequency and effects of contact with pharmaceutical reps
Madhavan[76]	1997	2	Knowledge and attitude of physicians
Magzoub[77]	2011	1	Factors influencing prescribing decisions of physicians
Mather[78]	2005	2	Attitudes and beliefs of physicians
McKinney[79]	1990	2	Attitudes of physicians

Monaghan[80]	2003	2	Knowledge and attitude of residents
Morgan[81]	2006	2	Opinions and practice patterns of doctors on acceptance and use of gifts from pharma
Okike[82]	2009	1	Accuracy of conflict-of-interest disclosures reported by physicians
Orlowski[83]	1992	4	Influence of interactions with pharma on physicians' prescribing patterns
Osborn[84]	2009	4	Effect of ethical guidelines on decision-making regarding the relationships with the pharma industry
Oshikoya[85]	2011	4	Drug information sources and their influence on drug prescribing behavior
Othman[86]	2010	2	Attitudes and beliefs of physicians towards information provided by pharmaceutical representatives
Pedan[87]	2011	4	Influence of interactions with pharma on physicians' prescribing patterns
Pinto[88]	2007	1	Physicians' intent to comply with the AMA's guidelines on gifts from the pharma industry
Prosser[89]	2003	1	Prescribing GPs' attitudes and approach, use of the pharma industry as a significant info source
Prosser[90]	2003	2	Beliefs of physicians
Prosser[91]	2003	4	Factors influencing prescribing patterns
Quinn[92]	2002	2	Attitude of physicians
Rajan[93]	2008	2	Beliefs and attitudes of physicians
Rohra[94]	2006	4	Evaluation of drug promotional claims made by pharma
Ross[95]	2009	2	Attitudes and beliefs of physicians
Roy[96]	2007	1	Range of drug promotional practices

Rutledge[97]	2003	2	Beliefs of physicians
Saito[98]	2010	2	Knowledge and attitudes of physicians
Sara[99]	2001	2	Attitudes and beliefs of physicians
Seidel[100]	1999	2	Pharma-physician interaction
Sharma[101]	2010	2	Attitudes of residents
Siddiqi[102]	2011	4	Influence of pharma on drug prescription
Skoglund[103]	2011	2	Attitudes and beliefs of physicians
Somerset[104]	2001	1	Pharma-physicians interactions - Goffman's dramaturgical model
Spiller[105]	2001	2	Attitudes and beliefs of physicians
Steinman[106]	2001	2	Attitude of residents towards pharma-industry promotions
Stelfox [107]	1998	4	Effects of financial conflict of interests
Stoddard[108]	2003	2	Attitudes and beliefs of physicians
Stross [109]	1987	2	Attitudes and beliefs of physicians
Tabas [110]	2011	2	Attitudes of physicians towards commercial support of CME
Taylor[111]	1991	4	Factors influencing prescribing patterns
Tengilimogclu [112]	2004	1	Ethical dilemmas faced by pharma sales rep in their relations with physicians
Thomas[113]	2005	2	Self-regulation and the relationship of physicians with the pharmaceutical industry.
Thomson[114]	1994	2	Attitudes of physicians towards pharmaceutical companies
Tobin[115]	2008	4	Influence of pharma on drug prescription
Trevisol[116]	2001	2	Attitudes and beliefs of physicians and residents towards drug advertisement
Weinfort, [117]	2006	2	Elicit the perspectives of research participants to help inform the development of appropriate guidance on disclosure of financial interests in research"

1. Aasland OG, Forde R: **[Physicians and drug industry: attitudes and practice].** *Tidsskrift for Den Norske Laegeforening* 2004, **124:**2603-2606.
2. Al-Areefi MA, Hassali MA, Mohamed Ibrahim MI: **The role of pharmaceutical marketing and other factors in prescribing decisions: the Yemeni experience.** *Res Social Adm Pharm* 2013, **9:**981-988.
3. Alghasham AA: **Generic drug prescribing in central Saudi Arabia: perceptions and attitudes of physicians.** *Annals of Saudi Medicine* 2009, **29:**24-29.

4. Alssageer MA, Kowalski SR: **A survey of pharmaceutical company representative interactions with doctors in Libya.** *Libyan J Med* 2012, **7**.
5. Al Zahrani H: **The impact of pharmaceutical promotions on primary health care physician's prescribing behaviour in KAMC in central region.** *Int J Med Sci Public Health* 2014, **3:**358-361.
6. Andaleeb SS, Tallman RF: **Physician attitudes toward pharmaceutical sales representatives.** *Health Care Management Review* 1995, **20:**68-76.
7. Anderson BL, Silverman GK, Loewenstein GF, Zinberg S, Schulkin J: **Factors associated with physicians' reliance on pharmaceutical sales representatives.** *Academic Medicine* 2009, **84:**994-1002.
8. Arkinson J, Holbrook A, Wiercioch W: **Public perceptions of physician-pharmaceutical industry interactions: A systematic review.** *Healthcare Policy* 2010, **5:**69-89.
9. Backer EL, Lebsack JA, Van Tonder RJ, Crabtree BF: **The value of pharmaceutical representative visits and medication samples in community-based family practices.** *Journal of Family Practice* 2000, **49:**811-816.
10. Banks JW, 3rd, Mainous AG, 3rd: **Attitudes of medical school faculty toward gifts from the pharmaceutical industry.** *Academic Medicine* 1992, **67:**610-612.
11. Berings D, Blondeel L, Habraken H: **The effect of industry-independent drug information on the prescribing of benzodiazepines in general practice.** *Eur J Clin Pharmacol* 1994, **46:**501-505.
12. Brett AS, Burr W, Moloo J: **Are gifts from pharmaceutical companies ethically problematic? A survey of physicians.** *Archives of Internal Medicine* 2003, **163:**2213-2218.
13. Burashnikova IS, Ziganshin AU, Ziganshina LE: **Attitudes to pharmaceutical promotion techniques among healthcare professionals in the Republic of Tatarstan, Russia.** *International Journal of Risk and Safety in Medicine* 2008, **20:**57-71.
14. Buusman A, Andersen M, Merrild C, Elverdam B: **Factors influencing GPs' choice between drugs in a therapeutic drug group. A qualitative study.** *Scandinavian Journal of Primary Health Care* 2007, **25:**208-213.
15. Caamano F, Figueiras A, Gestal-Otero JJ: **Influence of commercial information on prescription quantity in primary care.** *European Journal of Public Health* 2002, **12:**187-191.
16. Campo K, De Staebel O, Gijsbrechts E, van Waterschoot W: **Physicians' decision process for drug prescription and the impact of pharmaceutical marketing mix instruments.** *Health Marketing Quarterly* 2005, **22:**73-107.
17. Carroll AE, Vreeman RC, Buddenbaum J, Inui TS: **To what extent do educational interventions impact medical trainees' attitudes and behaviors regarding industry-trainee and industry-physician relationships?** *Pediatrics* 2007, **120:**e1528-1535.
18. Carthy P, Harvey I, Brawn R, Watkins C: **A study of factors associated with cost and variation in prescribing among GPs.** *Family Practice* 2000, **17:**36-41.
19. Castresana L, Mejia R, Aznar M: **[The attitude of physicians regarding the promotion strategies of the pharmaceutical industry].** *Medicina* 2005, **65:**247-251.
20. Caudill TS, Johnson MS, Rich EC, McKinney WP: **Physicians, pharmaceutical sales representatives, and the cost of prescribing.** *Archives of Family Medicine* 1996, **5:**201-206.
21. Chimonas S, Brennan TA, Rothman DJ: **Physicians and drug representatives: exploring the dynamics of the relationship.** *Journal of General Internal Medicine* 2007, **22:**184-190.
22. Chren MM, Landefeld CS: **Physicians' behavior and their interactions with drug companies. A controlled study of physicians who requested additions to a hospital drug formulary.** *JAMA* 1994, **271:**684-689.
23. Civaner M: **Sale strategies of pharmaceutical companies in a "pharmerging" country: The problems will not improve if the gaps remain.** *Health Policy* 2012, **106:**225-232.

24. Creyer EH, Hrsistodoulakis I: **Marketing pharmaceutical products to physicians. Sales reps influence physicians' impressions of the industry.** *Marketing Health Services* 1998, **18:**34-38.
25. De Las Cuevas C, Sanz EJ, De La Fuente JA: **Variations in antidepressant prescribing practice: clinical need or market influences?** *Pharmacoepidemiology & Drug Safety* 2002, **11:**515-522.
26. de Gara CJ, Rennick KC, Hanson J: **Perceptions of conflict of interest: surgeons, internists, and learners compared.** *Am J Surg* 2013, **205:**541-545; discussion 545-546.
27. Ellison JA, Hennekens CH, Wang J, Lundberg GD, Sulkes D: **Low Rates of Reporting Commercial Bias by Physicians Following Online Continuing Medical Education Activities.** *American Journal of Medicine* 2009, **122:**875-878.
28. Evans DV, Hartung DM, Andeen G, Mahler J, Haxby DG, Kraemer DF, Fagnan LJ: **One practice's experiment in refusing detail rep visits.** *Journal of Family Practice* 2011, **60:**E1-6.
29. Evans KR, Beltramini RF: **Physician acquisition of prescription drug information.** *Journal of Health Care Marketing* 1986, **6:**15-25.
30. Fagundes MJ, Soares MG, Diniz NM, Pires JR, Garrafa V: **[Bioethical analysis of drugs advertisement and publicity].** *Ciencia & Saude Coletiva* 2007, **12:**221-229.
31. Fassold RW, Gowdey CW: **A survey of physicians' reactions to drug promotion.** *Canadian Medical Association Journal* 1968, **98:**701-705.
32. Diaz Gravalos GJ, Palmeiro Fernandez G, Nunez Masid E, Casado Gorriz I: **Opinion of the primary care physicians of Ourense with respect to certain aspects of their prescription of medicines. [Spanish]**

Opinion de los medicos de atencion primaria de Ourense sobre algunos aspectos de su prescripcion farmaceutica. *Revista Espanola de Salud Publica* 2001, **75:**361-371.
33. Figueiras A, Caamano F, Gestal-Otero JJ: **Influence of physician's education, drug information and medical-care settings on the quality of drugs prescribed.** *European Journal of Clinical Pharmacology* 2000, **56:**747-753.
34. Fischer MA, Keough ME, Baril JL, Saccoccio L, Mazor KM, Ladd E, Von Worley A, Gurwitz JH: **Prescribers and pharmaceutical representatives: why are we still meeting?** *Journal of General Internal Medicine* 2009, **24:**795-801.
35. Fortuna RJ, Ross-Degnan D, Finkelstein J, Zhang F, Campion FX, Simon SR: **Clinician attitudes towards prescribing and implications for interventions in a multi-specialty group practice.** *Journal of Evaluation in Clinical Practice* 2008, **14:**969-973.
36. Freeman JK, Barnes JH, Summers KH, Szeinbach SL: **Modeling physicians' prescribing decisions for patients with panic disorder.** *Journal of Health Care Marketing* 1993, **13:**34-39.
37. Fugh-Berman A, McDonald CP, Bell AM, Bethards EC, Scialli AR: **Promotional tone in reviews of menopausal hormone therapy after the Women's Health Initiative: an analysis of published articles.** *PLoS Med* 2011, **8:**e1000425.
38. Gaedeke RM, Tootelian DH, Sanders EE: **Value of services provided by pharmaceutical companies: perceptions of physicians and pharmaceutical sales representatives.** *Health Marketing Quarterly* 1999, **17:**23-31.
39. Gaither CA, Bagozzi RP, Kirking DM, Ascione FJ: **Factors related to physicians' attitudes and beliefs toward drug information sources.** *Drug Information Journal* 1994, **28:**817-827.
40. Gaither CA, Bagozzi RP, Ascione FJ, Kirking DM: **A reasoned action approach to physicians' utilization of drug information sources.** *Pharmaceutical Research* 1996, **13:**1291-1298.
41. Guloksuz S, Oral ET, Ulas H: **[Attitudes and behaviors of psychiatry residents and psychiatrists working in training institutes towards the relationship between the pharmaceutical industry and physicians].** *Turk Psikiyatri Derg* 2009, **20:**236-242.

42. Gray SW, Hlubocky FJ, Ratain MJ, Daugherty CK: **Attitudes toward research participation and investigator conflicts of interest among advanced cancer patients participating in early phase clinical trials.** *Journal of Clinical Oncology* 2007, **25:**3488-3494.
43. Guldal D, Semin S: **The influences of drug companies' advertising programs on physicians.** *International Journal of Health Services* 2000, **30:**585-595.
44. Gundermann C, Meier-Hellmann A, Bauer M, Hartmann M: **[Effects of a mandatory guideline that prohibit hospital doctors from accepting any form of benefits in any form from the pharmaceutical industry].** *Deutsche Medizinische Wochenschrift* 2010, **135:**67-70.
45. Gunnarsdottir AI, Kinnear M: **Factors that influence prescribers in their selection and use of COX-2 selective inhibitors as opposed to non-selective NSAIDs.** *Pharm World Sci* 2005, **27:**316-320.
46. Haayer F: **Rational prescribing and sources of information.** *Social Science & Medicine* 1982, **16:**2017-2023.
47. Hajjaj FM, Salek MS, Basra MK, Finlay AY: **Nonclinical influences, beyond diagnosis and severity, on clinical decision making in dermatology: understanding the gap between guidelines and practice.** *British Journal of Dermatology* 2010, **163:**789-799.
48. Hall KB, Tett SE, Nissen LM: **Perceptions of the influence of prescription medicine samples on prescribing by family physicians.** *Medical Care* 2006, **44:**383-387.
49. Halperin EC, Hutchison P, Barrier RC, Jr.: **A population-based study of the prevalence and influence of gifts to radiation oncologists from pharmaceutical companies and medical equipment manufacturers.** *International Journal of Radiation Oncology, Biology, Physics* 2004, **59:**1477-1483.
50. Hampson LA, Agrawal M, Joffe S, Gross CP, Verter J, Emanuel EJ: **Patients' views on financial conflicts of interest in cancer research trials.** *N Engl J Med* 2006, **355:**2330-2337.
51. Hassali MA, Kong DCM, Stewart K: **Generic medicines: Perceptions of general practitioners in Melbourne, Australia.** *Journal of Generic Medicines* 2006, **3:**214-225.
52. Hayes TM, Allery LA, Harding KG, Owen PA: **Continuing education for general practice and the role of the pharmaceutical industry.** *British Journal of General Practice* 1990, **40:**510-512.
53. Galan Herrera S, Delgado Marroquin MT, Altisent Trota R: **[Analysis of the relationship between primary care doctors and the pharmaceutical industry].** *Atencion Primaria* 2004, **34:**231-237.
54. Hodges B: **Interactions with the pharmaceutical industry: experiences and attitudes of psychiatry residents, interns and clerks.** *CMAJ Canadian Medical Association Journal* 1995, **153:**553-559.
55. Hull FM, Marshall T: **Sources of information about new drugs and attitudes towards drug prescribing: an international study of differences between primary care physicians.** *Family Practice* 1987, **4:**123-128.
56. Jones MI, Greenfield SM, Bradley CP: **Prescribing new drugs: qualitative study of influences on consultants and general practitioners.** *BMJ* 2001, **323:**378-381.
57. Jones MI, Greenfield SM, Bradley CP, Jowett S: **Prescribing new drugs: A survey of hospital consultants in the West Midlands.** *International Journal of Pharmacy Practice* 2000, **8:**285-290.
58. Joubert PH, Skene D: **Attitudes of private medical practitioners towards package inserts and other drug information sources.** *South African Medical Journal Suid-Afrikaanse Tydskrif Vir Geneeskunde* 1984, **66:**306-307.
59. Kao AC, Braddock C, 3rd, Clay M, Elliott D, Epstein SK, Filstead W, Hotze T, May W, Reenan J: **Effect of educational interventions and medical school policies on medical students' attitudes toward pharmaceutical marketing practices: a multi-institutional study.** *Academic Medicine* 2011, **86:**1454-1462.

60. Katz HP, Goldfinger SE, Fletcher SW: **Academia-industry collaboration in continuing medical education: description of two approaches.** *Journal of Continuing Education in the Health Professions* 2002, **22:**43-54.
61. Keim SM, Mays MZ, Grant D: **Interactions between emergency medicine programs and the pharmaceutical industry.** *Academic Emergency Medicine* 2004, **11:**19-26.
62. Kelly MH, Murray TS: **Motivation of general practitioners attending postgraduate education.** *British Journal of General Practice* 1996, **46:**353-356.
63. Klemenc-Ketis Z, Kersnik J: **The assessment of pharmaceutical sales representatives by family physicians--does it affect the prescribing index?** *Fam Pract* 2013, **30:**320-324.
64. Kerridge I, Maguire J, Newby D, McNeill PM, Henry D, Hill S, Day R, Macdonald G, Stokes B, Henderson K: **Cooperative partnerships or conflict-of-interest? A national survey of interaction between the pharmaceutical industry and medical organizations.** *Internal Medicine Journal* 2005, **35:**206-210.
65. Kersnik J, Klemenc-Ketis Z, Petek-Ster M, Tusek-Bunc K, Poplas-Susic T, Kolsek M: **Family doctors' views of pharmaceutical sales representatives: assessment scale development.** *Family Practice* 2011, **28:**456-460.
66. Keune JD, Vig S, Hall BL, Matthews BD, Klingensmith ME: **Taking disclosure seriously: disclosing financial conflicts of interest at the American College of Surgeons.** *Journal of the American College of Surgeons* 2011, **212:**215-224.
67. Kim SY, Millard RW, Nisbet P, Cox C, Caine ED: **Potential research participants' views regarding researcher and institutional financial conflicts of interest.** *J Med Ethics* 2004, **30:**73-79.
68. Kish-Doto J, Evans WD, Squire C, Williams P, Ranney LM, Melvin CL: **Patterns of prescribing antiepileptic drugs for bipolar disorder.[Erratum appears in J Psychiatr Pract. 2008 May;14(3):193].** *Journal of Psychiatric Practice* 2008, **14 Suppl 1:**35-43.
69. Klein D, MacDonald A, Drummond N, Cave A: **A qualitative study to identify factors influencing COXIB prescribed by family physicians for musculoskeletal disorders.** *Family Practice* 2006, **23:**659-665.
70. Korenstein D, Keyhani S, Ross JS: **Physician attitudes toward industry: a view across the specialties.** *Archives of Surgery* 2010, **145:**570-577.
71. Kyle GJ, Nissen LM, Tett SE: **Pharmaceutical company influences on medication prescribing and their potential impact on quality use of medicines.** *Journal of Clinical Pharmacy & Therapeutics* 2008, **33:**553-559.
72. Kyle GJ, Nissen LM, Tett SE: **The Australian rise of esomeprazole-was expenditure on samples a contributor?** *Pharmacoepidemiology & Drug Safety* 2009, **18:**62-68.
73. Lexchin J: **What information do physicians receive from pharmaceutical representatives?** *Canadian Family Physician* 1997, **43:**941-945.
74. Loh LC, Ong HT, Quah SH: **Impact of various continuing medical education activities on clinical practice - a survey of Malaysian doctors on its perceived importance.** *Annals of the Academy of Medicine, Singapore* 2007, **36:**281-284.
75. Lurie N, Rich EC, Simpson DE, Meyer J, Schiedermayer DL, Goodman JL, McKinney WP: **Pharmaceutical representatives in academic medical centers: interaction with faculty and housestaff.** *Journal of General Internal Medicine* 1990, **5:**240-243.
76. Madhavan S, Amonkar MM, Elliott D, Burke K, Gore P: **The gift relationship between pharmaceutical companies and physicians: an exploratory survey of physicians.** *Journal of Clinical Pharmacy & Therapeutics* 1997, **22:**207-215.
77. Magzoub MA, Neyaz Y, Khoja T, Qureshi NA, Haycox A, Walley T: **Determinants of physicians' medication prescribing behaviour in primary care in Riyadh city, Saudi Arabia. [French]**

Mode de prescription de medicaments en soins de sante primaires a Riyad (Arabie saoudite). *Eastern Mediterranean Health Journal* 2011, **17:**160-166.
78. Mather C: **The pipeline and the porcupine: alternate metaphors of the physician-industry relationship.** *Social Science & Medicine* 2005, **60:**1323-1334.
79. McKinney WP, Schiedermayer DL, Lurie N, Simpson DE, Goodman JL, Rich EC: **Attitudes of internal medicine faculty and residents toward professional interaction with pharmaceutical sales representatives.** *JAMA* 1990, **264:**1693-1697.
80. Monaghan MS, Galt KA, Turner PD, Houghton BL, Rich EC, Markert RJ, Bergman-Evans B: **Student understanding of the relationship between the health professions and the pharmaceutical industry.** *Teaching & Learning in Medicine* 2003, **15:**14-20.
81. Morgan MA, Dana J, Loewenstein G, Zinberg S, Schulkin J: **Interactions of doctors with the pharmaceutical industry.** *Journal of Medical Ethics* 2006, **32:**559-563.
82. Okike K, Kocher MS, Wei EX, Mehlman CT, Bhandari M: **Accuracy of conflict-of-interest disclosures reported by physicians.** *New England Journal of Medicine* 2009, **361:**1466-1474.
83. Orlowski JP, Wateska L: **The effects of pharmaceutical firm enticements on physician prescribing patterns. There's no such thing as a free lunch.** *Chest* 1992, **102:**270-273.
84. Osborn M, Day R, Komesaroff P, Mant A: **Do ethical Guidelines make a difference to decision-making?** *Internal Medicine Journal* 2009, **39:**800-805.
85. Oshikoya KA, Oreagba IA, Adeyemi O: **Drug information resources and their influence on the prescribing behaviour of doctors in a teaching hospital in Nigeria.** *Pharmacoepidemiology and Drug Safety* 2011, **20:**S39-S40.
86. Othman N, Vitry AI, Roughead EE, Ismail SB, Omar K: **Medicines information provided by pharmaceutical representatives: a comparative study in Australia and Malaysia.** *BMC Public Health* 2010, **10:**743.
87. Pedan A, Wu H: **Asymmetric responsiveness of physician prescription behavior to drug promotion of competitive brands within an established therapeutic drug class.** *Health Marketing Quarterly* 2011, **28:**133-154.
88. Pinto SL, Lipowski E, Segal R, Kimberlin C, Algina J: **Physicians' intent to comply with the American Medical Association's guidelines on gifts from the pharmaceutical industry.** *Journal of Medical Ethics* 2007, **33:**313-319.
89. Prosser H, Almond S, Walley T: **Influences on GPs' decision to prescribe new drugs-the importance of who says what.** *Family Practice* 2003, **20:**61-68.
90. Prosser H, Walley T: **Understanding why GPs see pharmaceutical reprensentatives: A qualitative interview study.** *British Journal of General Practice* 2003, **53:**305-311.
91. Prosser H, Walley T: **New drug uptake: qualitative comparison of high and low prescribing GPs' attitudes and approach.** *Family Practice* 2003, **20:**583-591.
92. Quinn RJ, O'Neill C: **Attitudes of general practitioners to their interactions with pharmaceutical companies: a qualitative study.** *Irish Medical Journal* 2002, **95:**199-202.
93. Rajan MSV, Sreedhar, Khan SA, Thiyagu R, Rao PGM: **Information seeking behaviour of clinicians in a semi urban town in Southern India.** *Journal of Clinical and Diagnostic Research* 2008, **2:**1069-1073.
94. Rohra DK, Gilani AH, Memon IK, Perven G, Khan MT, Zafar H, Kumar R: **Critical evaluation of the claims made by pharmaceutical companies in drug promotional material in Pakistan.** *Journal of Pharmacy & Pharmaceutical Sciences* 2006, **9:**50-59.
95. Ross JS, Keyhani S, Korenstein D: **Appropriateness of collaborations between industry and the medical profession: physicians' perceptions.** *American Journal of Medicine* 2009, **122:**955-960.
96. Roy N, Madhiwalla N, Pai SA: **Drug promotional practices in Mumbai: a qualitative study.** *Indian Journal of Medical Ethics* 2007, **4:**57-61.

97. Rutledge P, Crookes D, McKinstry B, Maxwell SR: **Do doctors rely on pharmaceutical industry funding to attend conferences and do they perceive that this creates a bias in their drug selection? Results from a questionnaire survey.** *Pharmacoepidemiology & Drug Safety* 2003, **12:**663-667.
98. Saito S, Mukohara K, Bito S: **Japanese practicing physicians' relationships with pharmaceutical representatives: a national survey.** *PLoS ONE [Electronic Resource]* 2010, **5:**e12193.
99. Sara G, Prior N: **Views of psychiatrists and trainees on drug company involvement in CME activities.** *Australasian Psychiatry* 2001, **9:**101-105.
100. Seidel WF: **The GP, the rep and the handouts.** *South African Medical Journal Suid-Afrikaanse Tydskrif Vir Geneeskunde* 1999, **89:**520-523.
101. Sharma V, Aggarwal S, Singh H, Garg S, Sharma A, Sharma R: **Attitudes and practices of medical graduates in Delhi towards gifts from the pharmaceutical industry.** *Indian Journal of Medical Ethics* 2010, **7:**223-225.
102. Siddiqi A, Hussain S, Parveen G, Malik F, Yasin F, Akram TS, Hameed A, Riaz H, Shah PA, Saeed T: **Relevant influence of promotional tools by pharmaceutical industry on prescribing behaviors of doctors: A cross-sectional survey in Pakistan.** *African Journal of Pharmacy and Pharmacology* 2011, **5:**1623-1632.
103. Skoglund I, Bjorkelund C, Mehlig K, Gunnarsson R, Moller M: **GPs' opinions of public and industrial information regarding drugs: a cross-sectional study.** *BMC Health Services Research* 2011, **11:**204.
104. Somerset M, Weiss M, Fahey T: **Dramaturgical study of meetings between general practitioners and representatives of pharmaceutical companies; Commentary: dramaturgical model gives valuable insight.** *BMJ* 2001, **323:**1481-1484.
105. Spiller LD, Wymer WW, Jr.: **Physicians' perceptions and uses of commercial drug information sources: an examination of pharmaceutical marketing to physicians.** *Health Marketing Quarterly* 2001, **19:**91-106.
106. Steinman MA, Shlipak MG, McPhee SJ: **Of principles and pens: attitudes and practices of medicine housestaff toward pharmaceutical industry promotions.** *American Journal of Medicine* 2001, **110:**551-557.
107. Stelfox HT, Chua G, O'Rourke K, Detsky AS: **Conflict of interest in the debate over calcium-channel antagonists.** *N Engl J Med* 1998, **338:**101-106.
108. Stoddard JJ, Reed M, Hadley J: **Financial incentives and physicians' perceptions of conflict of interest and ability to arrange medically necessary services.** *Journal of Ambulatory Care Management* 2003, **26:**39-50.
109. Stross JK: **Information sources and clinical decisions.** *Journal of General Internal Medicine* 1987, **2:**155-159.
110. Tabas JA, Boscardin C, Jacobsen DM, Steinman MA, Volberding PA, Baron RB: **Clinician attitudes about commercial support of continuing medical education: Results of a detailed survey.** *Archives of Internal Medicine* 2011, **171:**840-846.
111. Taylor RJ, Bond CM: **Change in the established prescribing habits of general practitioners: an analysis of initial prescriptions in general practice.** *British Journal of General Practice* 1991, **41:**244-248.
112. Tengilimoglu D, Kisa A, Ekiyor A: **The pharmaceutical sales rep/physician relationship in Turkey: ethical issues in an international context.** *Health Marketing Quarterly* 2004, **22:**21-39.
113. Thomas JM: **Self-regulation and the relationship of physicians with the pharmaceutical industry.** *Virtual Mentor* 2005, **7**.
114. Thomson AN, Craig BJ, Barham PM: **Attitudes of general practitioners in New Zealand to pharmaceutical representatives.** *British Journal of General Practice* 1994, **44:**220-223.

115. Tobin L, de Almedia Neto AC, Wutzke S, Patterson C, Mackson J, Weekes L, Williamson M: **Influences on the prescribing of new drugs.** *Australian Family Physician* 2008, **37:**78-80, 83.
116. Trevisol DJ, Ferreira MBC, Karnopp ZMP: **Drug advertisement in a medicine school in the Southern of Brazil. [Portuguese]**

A propaganda de medicamentos em escola de medicina do Sul do Brasil. *Ciencia e Saude Coletiva* 2010, **15:**3487-3496.
117. Weinfurt KP, Friedman JY, Allsbrook JS, Dinan MA, Hall MA, Sugarman J: **Views of potential research participants on financial conflicts of interest: barriers and opportunities for effective disclosure.** *J Gen Intern Med* 2006, **21:**901-906.
