# Supplementary material for: Knowledge, Beliefs and Attitudes of Patients and the General Public towards the Interactions of Physicians with the Pharmaceutical and the Device Industry: A Systematic Review
Source: PLoS One. 2016 Aug 24;11(8):e0160540. doi: 10.1371/journal.pone.0160540 (PMC4996522; doi:10.1371/journal.pone.0160540)
Supplement: S1 Table — (PDF) [file pone.0160540.s004.pdf]

S1Table. Methodological features of included studies on interactions with the pharmaceutical industry

a. Methodological features for survey studies

**Study ID**	**Sample size calculation**	**Sampling:**<br>•**Frame**<br>•**Method (type)**	**Recruitment method**	**Respons e rate**	**Administration method**	**Validity of tool**<br>**Pilot testing done**
Blake, 1995[1]	Not reported	•Frame: Adults (I8 years of age and older) in two family practice centers operated by the University of Missouri-Columbia, Department of Family and Community Medicine.<br>•Method: Convenient sampling	In-person	83.1%	In person self-administered questionnaire	•Validity of tool: not reported<br>•Pilot-testing: Yes<br>"The questionnaire was developed specifically for this study and was pilot-tested on 43 adults in the waiting room of the Columbia clinic."
La puma, 1995[2]	Not reported	•Frame: Patients 18 years and above in a general medical office.<br>•Method: Convenient sampling questionnaire distributed by a research associate to consecutive patients	In person	74%	In person, self-administered questionnaire.	•Validity of tool: Valid<br>•Pilot-testing: Yes "The questionnaire for patients was pretested and validated"
Mainous, 1995[3]	Not reported	•Frame: Data base of phone numbers<br>•"an annual statewide omnibus survey of adult (18 years of age and older) Kentucky residents"<br>•Method: stratified random sampling	Telephone surveys "Waksberg clustering supplies an almost completely unbiased sample of households with telephones."	55%	Telephone surveys	•Validity of tool: not reported<br>•Pilot-testing: not reported
Gibbons, 1998[4]	Not reported	•Frame: Patients at two tertiary-care medical centers, one military and one civilian, at Washington, DC.<br>•Method: Random sampling at the military center, convenient sampling at the civilian center.	Not reported, probably in person.	96% at the military center; not applicabl	In person, interviewer administered	•Self-developed tool, no validation reported.<br>•Pilot-testing: Yes. "The survey was pilot tested to ensure clarity and understanding".

				e at the civilian center		
Qidawai, 2003[5]	Not reported	•Frame: Patients attending the outpatient settings of a busy tertiary care hospital<br>•Method: Convenient sampling	In person<br>"The questionnaire was administered by the study authors and trained volunteers."	Not reported	Self-administered questionnaires<br>"The questionnaire was administered by the study authors and trained volunteers"	•Validity of tool: not reported<br>Pilot-testing: Not reported
Semin, 2006[6]	Yes	•Frame: Patients who had been admitted to the primary health care centers in Izmir Centrum, the third largest city in Turkey.<br>•Method: Stratified systematic sampling<br>"44 primary health care centers were chosen among the 112 located in the city center"	In person<br>"Almost all of the patients who were asked to participate in the study accepted it"	Not reported	In-person, self-administered questionnaires	•Validity of tool: not reported<br>•Pilot testing: not reported
Edwards, 2009[7]	Not reported	•Frame: employees of The Age newspaper in Melbourne. N=1524<br>•Method: Convenient sampling	E-mail	8.8%	Internet<br>"The survey was distributed through a bulk email containing a link to the website Survey Monkey"	•Validity of tool: Not reported<br>•Pilot testing: Yes<br>"The internal consistency of the questionnaire was good, as measured by Cronbach's alpha: 0.78"
Jastifer, 2009[8]	Not reported	•Frame: List of postal addresses Adult residents, 18 years and older, who reside in Alger County, in rural Michigan."<br>•Method: Convenience sampling<br>"The survey was mailed to every residential postal address in Alger County."	Mail<br>"Two copies of the survey, consent forms, and a letter explaining the survey were mailed to every residential postal address in Alger County."	10.1%	Mail<br>self-administered questionnaires	•Validity of tool: Not reported<br>•Pilot-testing: Not reported

Tattersall, 2009[9]	Yes	• Frame: Three general practices in metropolitan Sydney<br>• Method: Convenient sampling: "During October–November 2007, we surveyed patients attending three general practices in metropolitan Sydney, New South Wales.	In person<br>"Patients were either approached in the waiting room by a researcher or invited to participate by the doctor."	80%	In person, self-administered questionnaire	• Validity of tool: Not reported.<br>• Pilot-testing: Yes "In a pilot study, 223 patients attending a rural NSW practice received the survey" "A split-half reliability analysis of the weighted data revealed a Cronbach's α of 0.91."
Macneill, 2010[10]	Not reported	• Frame: Electoral roll of the Hunter region of New South Wales Adults of New South Wales over the age of 18 years<br>• Method: Random sampling: "The public survey was mailed to 3000 people over the age of 18 years randomly sampled from the electoral roll of the Hunter region of New South Wales."	Mail<br>"The public survey was mailed to 3000 people over the age of 18 years"	26%	Mail	• Validity of tool: Not reported<br>• Pilot-testing: not reported
Grande, 2012[11]	Not reported but the objective was to complete interviews on 30 African American and 30 White respondents in each MSA.	• Frame: A database of phone numbers "African-American and White adults in 40 large metropolitan areas"<br>• Method: Cluster random sampling. "A random sample of land-line telephone numbers from each Metropolitan Statistical Areas was selected."	Phone<br>"a random digit dial telephone survey of African American and White non-Latino adults in 40 metropolitan areas"	31.1%	Computer assisted telephone interview	• Validity of tool: Previously reported validated too<br>• Pilot testing: Not reported
Green, 2012[12]	Not calculated but authors mentioned they wanted to reach 200 individuals	• Frame: patients in waiting rooms of five outpatient clinics at a mid-Atlantic academic medical center. Eligible participants included all English-speaking adults (>18 years old) in the waiting areas of five	In person<br>"A fourth-year medical student explained the purpose of the survey and obtained verbal	87%	In-person, self-administered questionnaires (with assistance)<br><br>61-item survey	• Validity of tool: Previously reported validated tool<br>• Pilot-testing: Yes "These were then pilot tested for face and

		clinics (Primary Care (two), Derma-tology, Orthopedics, and Ophthalmology).<br>•Method: Convenient Sampling	consent from potential participants”			content validity with a convenience sample of patients, physicians, and the general public, then modified in response to feedback”
Wise, 2012[13]	Not reported	•Frame: Postoperative adult patients at Grey's Hospital, Pietermaritzburg.<br>•Method: Convenience sampling	In-person<br>“A sample of postoperative patients was taken from four surgical wards”	Not reported	In-person, self-administered questionnaires	•Validity of tool: not reported<br>•Pilot-testing: not reported
Camp, 2013[14]	Not reported	•Frame: postoperative patients attending follow up hip and knee arthroplasty clinics at Mount Sinai Hospital and Holland Orthopaedic , Arthritic Centre and the New England Baptist Hospital in US(1) and Canada (2)<br>English-speaking patients who were at least eighteen years old and who had undergone primary or revision hip or knee arthroplasty at least three months earlier were eligible to participate in the study.<br>•Method: Convenient sampling	In person-<br>“Patients attending follow-up arthroplasty clinics at the participating hospitals were asked by clinic personnel or the first author”	88% for US, 92% for Canada, Combine d rate: 90%	In-person, self-administered questionnaires (with no assistance)<br><br>40 item questionnaire (3-likert scale):	•Validity of tool: Not reported<br>•Pilot testing: Yes “Testing the derived questionnaire on nonsurgical volunteers and postoperative arthroplasty patients in addition to using cognitive interviews to ensure that questions would be understood.”

Holbrook, 2013[15]	Not reported but authors mentioned the target was 1000 Canadian residents to allow the proportions to be estimated with an accuracy of ±3%, with 95% confidence	•Frame: A database of phone numbers<br>Adult population of Canada (adults 18 years of age or older who speak English or French), and reside in private homes<br>•Method: Stratified random sampling "· All 10 provinces, but not the territories, were included, with representation in proportion to population."<br>.	Telephone interview "Random digit dial procedures were utilized to select households across Canada and, within households; the most recent birthday selection method used"	46.8%	Telephone survey	•Validity of tool: Yes "Tool was previously developed based on a systematic review of the literature, COI guidelines and consultation with COI bilingual translation experts and validated using a series of cognitive interviews"<br>•Pilot-testing: Not reported

b. Checklist for methodological quality of included qualitative studies

Author's last name, publication year (SN)	Was there a clear statement of the aims of the research?	Is a qualitative methodology appropriate?	Was the research design appropriate to address the aims of the research?	Was the recruitment strategy appropriate to the aims of the research?	Was the data collected in a way that addressed the research issue?	Has the relationship between researcher and participants been adequately considered?	Have ethical issues been taken into consideration?	Was the data analysis sufficiently rigorous?	Is there a clear statement of findings?	How valuable is the research?
Oakes, 2015[16]	Y	Y	Y	Y	Y	N	Y	?	Y	Y

References:

1. Blake RL, Jr., Early EK: **Patients' attitudes about gifts to physicians from pharmaceutical companies**. *The Journal of the American Board of Family Practice / American Board of Family Practice* 1995, **8**(6):457-464.
2. La Puma J, Stocking CB, Rhoades WD, Darling CM, Ferner RE, Neuberger J, VandenBurg M, Dews I, Tobias JS: **Financial ties as part of informed consent to postmarketing research. Attitudes of American doctors and patients**. *Bmj* 1995, **310**(6995):1660-1663.
3. Mainous AG, 3rd, Hueston WJ, Rich EC: **Patient perceptions of physician acceptance of gifts from the pharmaceutical industry**. *Archives of family medicine* 1995, **4**(4):335-339.
4. Gibbons RV, Landry FJ, Blouch DL, Jones DL, Williams FK, Lucey CR, Kroenke K: **A comparison of physicians' and patients' attitudes toward pharmaceutical industry gifts**. *Journal of general internal medicine* 1998, **13**(3):151-154.
5. Qidawai WQ, H.; Sohail, S.; Alam, M.; Azam, S.: **PERCEPTIONS ON BIOETHICS AMONG PATIENTS PRESENTING TO FAMILY PHYSICIANS AT A TEACHING HOSPITAL IN KARACHI, PAKISTAN**. *Pak J Med Sci* 2003, **19**(3):192-196.
6. Semin S, Guldal D, Ozcakar N, Mevsim V: **What patients think about promotional activities of pharmaceutical companies in Turkey**. *Pharmacy world & science : PWS* 2006, **28**(4):199-206.
7. Edwards D, Ballantyne A: **Patient awareness and concern regarding pharmaceutical manufacturer interactions with doctors**. *Internal medicine journal* 2009, **39**(3):191-196.
8. Jastifer J, Roberts S: **Patients' awareness of and attitudes toward gifts from pharmaceutical companies to physicians**. *International journal of health services : planning, administration, evaluation* 2009, **39**(2):405-414.
9. Tattersall MH, Dimoska A, Gan K: **Patients expect transparency in doctors' relationships with the pharmaceutical industry**. *The Medical journal of Australia* 2009, **190**(2):65-68.
10. Macneill PU, Kerridge IH, Newby D, Stokes BJ, Doran E, Henry DA: **Attitudes of physicians and public to pharmaceutical industry 'gifts'**. *Internal medicine journal* 2010, **40**(5):335-341.
11. Grande D, Shea JA, Armstrong K: **Pharmaceutical industry gifts to physicians: patient beliefs and trust in physicians and the health care system**. *Journal of general internal medicine* 2012, **27**(3):274-279.
12. Green MJ, Masters R, James B, Simmons B, Lehman E: **Do gifts from the pharmaceutical industry affect trust in physicians?** *Family medicine* 2012, **44**(5):325-331.
13. R. WRR: **The opinion of patients at a local South African teaching hospital on physician - industry relations**. *South Afr J Anaesth Analg* 2013, **19**(3):160-163.
14. Camp MW, Mattingly DA, Gross AE, Nousiainen MT, Alman BA, McKneally MF: **Patients' views on surgeons' financial conflicts of interest**. *The Journal of bone and joint surgery American volume* 2013, **95**(2):e9 1-8.
15. Holbrook A, Lexchin J, Pullenayegum E, Campbell C, Marlow B, Troyan S, Weijer C, Blackmer J, Brazil K, Willison D: **What do Canadians think about physician-pharmaceutical industry interactions?** *Health policy* 2013, **112**(3):255-263.
16. Mueller DH, Lungu D, Acharya A, Palmer N: **Constraints to implementing the Essential Health Package in Malawi**. *PLoS ONE* 2011, **6**(6):e20741.
