# Supplementary material for: Knowledge, Beliefs and Attitudes of Patients and the General Public towards the Interactions of Physicians with the Pharmaceutical and the Device Industry: A Systematic Review
Source: PLoS One. 2016 Aug 24;11(8):e0160540. doi: 10.1371/journal.pone.0160540 (PMC4996522; doi:10.1371/journal.pone.0160540)
Supplement: S2 Table — (PDF) [file pone.0160540.s005.pdf]

S2 Table: Summary of findings on interactions with the pharmaceutical industry

Study ID/	Participants and settings	Results
**Blake, 1995[1]**<br><br>Funding: not reported	• Adults (I8 years of age and older) in two family practice centers<br>• Columbia, USA; June and July 1994<br>• N = 486; 63.2% females; mean age (SD): 40.6 (±15.8)<br>• Education: 48.4% some college or college graduate; 17.1% postgraduate degree	**Awareness of the interactions of physicians in general:**<br>- Patient awareness of gifts sometimes provided to physicians by pharmaceutical companies: drug samples (87%); ballpoint pens (55%); medical books (35%); baby formula (29%); dinner (22%); coffee maker (14%).<br>**Beliefs about their effects on prescription behavior and quality of care:**<br>- Frequency of the influence of gifts from drug companies over a physician's prescription of medication in respondents' opinion: never (6.2%); rarely (18.3%); sometimes (53.9%); frequently (16%).<br>**Beliefs about their effects on cost of care:**<br>- 64% believed that gifts from drug companies to physicians increase the cost of medications.<br>Strong association between beliefs that gifts influence physicians' prescribing behavior and that gifts affect cost of care.<br>**Attitudes towards the interactions:**<br>- Approval of the following gifts from pharmaceutical companies to physicians: dinner (34.6%); baby formula (41.4%); cocktail party (40.5%); golf tournament (40.3%); coffee maker (39.1%); conference expenses (52.7%); ice cream social (55.6%); ballpoint pens (67.3%); medical books (70.0%); drug samples (82.1%).
**La Puma, 1995[2]**<br><br>Funding not reported	• Patients (18 years and above) in a general medical office<br>• USA<br>• N= 200; 64% females; mean age (range): 49.7 (18 to 87 years)	**Beliefs about their effects on prescription behavior and quality of care:**<br>- 69% of patients thought that some doctors might be influenced to enroll patients just for the fee.<br>**Attitudes towards the interactions:**<br>- 56% of patients found it unacceptable for doctors to receive a fee for taking part in post-market research<br>**Attitudes towards possible ways to manage the interactions:**<br>- 86% believed that a physician should inform a patient if the physician is paid for enrolling the patient.<br>- Patients who believed that doctors should tell patients: what company, agency, or foundation is paying for the study (85%); whether he or she owns stock in the sponsoring company (74%); whether he or she is paid a salary by the sponsoring company (78%); whether he or she is paid a fee for each patient enrolled (75%).
**Mainous, 1995[3]**<br><br>Funding not reported	• Kentucky residents (18 years of age and older)<br>• Kentucky, USA<br>• N= 649; 55% females; mean age (SD): 47± 16<br>• Education: 72% high school or above	**Awareness of the interactions of physicians in general:**<br>- Respondents who were aware that physicians received: gifts with a possible patient benefit (82%); personal gifts (32%).<br>**Attitudes towards the interactions:**<br>- 59% of respondents believed that physicians could accept as much office-use gifts as offered<br>- 33% of respondents believed that physicians could accept as much personal gifts as offered and 32% believed that personal gifts should be limited to less than $25 per year.<br>**Beliefs about their effects on prescription behavior and quality of care:**<br>- Respondents who believed that the following did not affect quality of care: office-use gifts (61%); personal gifts (54%).<br>**Beliefs about their effects on cost of care:**<br>- 38% of respondents believed that office-use gifts had no effect on cost of care; 26% believed it had negative effects.<br>- 42% of respondents believed that personal gifts had negative effect on cost of care; 30% believed it had no effect.

**Gibbons, 1998[4]**<br><br>Funding not reported	• Patients at two medical centers<br>• USA<br>• N= 100 at military site, 96 at civilian site; 65-67% female; mean age (range): 61 (21-89) at military site, 60 (24-90) at civilian site<br>• Education: college graduate: 29% at military site; 3.2% at civilian site	**Awareness of the interactions of own physicians:**<br>- 53% of patients were unsure whether or not their physicians accepted gifts.<br>**Awareness of the interactions of physicians in general:**<br>- 54% of patients were aware that pharmaceutical industry gifts were given to physicians.<br>- For patients who were unaware, 24% responded that this knowledge altered their perception of the profession.<br>**Attitudes towards the interactions:**<br>- Patients who thought gifts were not appropriate: trip (59%); dinner (47%); pocketknife (38%); lunch (23%); mug (23%); drug sample (22%); large text (20%); pen (19%); video (18%); small text (16%).<br>**Beliefs about their effects on prescription behavior and quality of care:**<br>- Patients who thought the following gifts influenced prescription behavior: trip (56%); dinner (48%); pocketknife (28%); lunch (29%); mug (31%); drug sample (42%); large text (38%); pen (31%); video (38%); small text (37%).<br>- 36% felt that acceptance of gifts compels a physician to prescribe products from that company.
**Qidawai, 2003[5]**<br><br>Funding not reported	• Patients attending outpatient tertiary care hospital<br>• Pakistan; December 1999 to May 2000<br>• N= 420; 11.2% females; mean Age (SD):33.7 (±11.98)<br>• Education: 34% graduate	**Attitudes towards the interactions:**<br>- 88% of the respondents agreed it is appropriate for doctors to accept gifts from pharmaceutical companies.
**Semin, 2006[6]**<br><br>A research grant from the Dokuz Eylul University, Turkey	• Patients admitted to the primary health care centers in Izmir Centrum<br>• Turkey; December 2004<br>• N= 584; 64.7% females; mean age (SD): 42 (± 15.4); 34.9% with chronic disease<br>• Education: 21% university	**Awareness of the interactions of physicians in general:**<br>- 82.7% were aware of pharmaceutical promotional activities.<br>**Beliefs about their effects on prescription behavior and quality of care:**<br>- 29.1% believed that physicians' drug choices were influenced by the gifts and ads of pharmaceutical companies.<br>- Participants who agreed that the following promotional activities influence prescription behavior: obtaining an electrocardiogram for the private office (74.1%); travels (69.9%); obtaining medical devices for the public primary health care center (70.9); invitation to conferences for the week-end at hotels (63.5%); dinner (62.8); conference and dinner (59.2%); middle level gifts like cover for the car seats (61.7%), invitation to congresses (52.9%); medical books (46.1%), small gifts (32.6%).<br>**Beliefs about their effects on cost of care:**<br>- 54.5% believed that promotion expenditures increase drug prices.<br>**Attitudes towards the interactions:**<br>- 55.3% put low reliability to the prescriptions of physicians who accept gifts from pharmaceutical companies.<br>- 71.2% agreed that accepting gifts from drug companies is not ethical.<br>- 82.5% supported the delivery of free samples that were given by pharmaceutical companies to people in need.<br>**Attitudes towards the effects of interactions on trust:**<br>-50% of patients stated they had low confidence in the prescriptions of physicians who accepted gifts from the pharmaceutical companies.<br>**Attitudes towards possible ways to manage the interactions:**<br>- 82% suggested that promotional activities aimed at doctors should be forbidden, restricted, or regulated.
**Edwards, 2009[7]**<br><br>Indirectly	• Employees of The Age newspaper in Melbourne	**Awareness of the interactions of physicians in general:**<br>- 40% were highly aware of pharmaceutical marketing.<br>**Beliefs about their effects on prescription behavior and quality of care:**

supported by the Donaghue Initiative at Yale University's Interdisciplinary Bioethics Center	• Australia; 18 January and 8 February 2007<br>• N= 134; 57.8% female; age: 40% 31-43, 34.1% 18-30, 25.3% 44-65<br>• Education: 37.6% Bachelor's degree; 14.5% postgraduate degree;	- 59% believed that pharmaceutical companies influenced doctors' prescriptions<br>**Attitudes towards the interactions**<br>- 39% reported that they would choose a doctor who did not see pharmaceutical representatives over one that would.<br>- 26% rated free drug samples as being appropriate, and 54.1% felt gifts irrelevant to medical practice were inappropriate<br>- Respondents who felt that the following interactions are highly untrustworthy: continuing medical education funded by the pharmaceutical industry (37%); promotional material provided by pharmaceutical sales representatives (43%); meeting with a pharmaceutical sales representative (50%).<br>- 50% felt that a meeting with a national prescribing service representative is highly trustworthy.<br>**Attitudes towards the effects of interactions on trust:**<br>-39% of employees reported that they would choose a doctor who did not see pharmaceutical representatives over one that would did. Those who believed information provided by the pharmaceutical industry to be inaccurate were significantly more likely to prefer a physician who did not receive promotional visits<br>**Attitudes towards possible ways to manage the interactions:**<br>- 48% would prefer to be informed about pharmaceutical marketing to some degree.<br>- 46% who wanted to be informed would prefer this to be through disclosure in the form of an accredited identification system, which indicated whether a doctor's practice receives promotional visits from pharmaceutical companies.
**Jastifer, 2009[8]**<br><br>Supported by Upper Peninsula Health Education Corporation, Michigan State University	• Adult residents (18 years and older) who reside in Alger County<br>• Michigan, USA<br>• N= 903; 63.1% females; age: 12.8% aged 18-40, 39.5% aged 41-60, 47.7% older than 60<br>• Education: 50.7% high-school graduate or some college; 34.8% college graduate or postgraduate degree	**Awareness of the interactions of physicians in general:**<br>- Patients' awareness of gifts to physicians from pharmaceutical companies: drug samples (94%); ballpoint pens (76%); medical books (38%); dinner out (37%); conference/travel expense (34%); spouse meal at dinner out (23%); golf tournament fees (19%).<br>**Beliefs about their effects on prescription behavior and quality of care:**<br>- 41.2% believed that receiving a gift from a drug company influenced prescription behavior.<br>**Beliefs about their effects on cost of care:**<br>- 67.3% believed that gifts to physicians from drug companies increased the cost of medications.<br>**Attitudes towards the interactions:**<br>- Patients who approved of specific gifts from pharmaceutical companies to physicians: drug samples (70%); ballpoint pens (54%); medical books (49%); dinner out (12%); conference/travel expense (14%); spouse meal at dinner out (7%); golf tournament fees (4%).<br>**Attitudes towards possible ways to manage the interactions:**<br>- 26.9% responded that physicians should disclose personal gifts received from drug companies.
**Tattersall, 2009[9]**<br><br>Funding not reported	• Patients in the waiting rooms of three general practices<br>• Australia; October to November 2007<br>• N= 906; 48.5% female; mean age (SD): 51.2 (±104.7)<br>• Education: 71.3% undergraduate or postgraduate university degree	**Awareness of the interactions of own physicians:**<br>- 76% were unaware of any competing interest their doctor may have with drug companies.<br>- 81% were unaware of benefits or financial incentives their doctor may obtain for prescribing a particular drug treatment<br>**Beliefs about their effects on prescription behavior and quality of care:**<br>- 49% believed that doctors are not unduly influenced despite receiving benefits or perks (27% disagreed).<br>**Attitudes towards possible ways to manage the interactions:**<br>- An average of 79% wanted to know about any incentives obtained by the doctor.<br>- Percentage of patients who would like to know if their doctor has: obtained any benefits in cash or in kind (71%); received or is receiving financial incentives for participation in research activities (69%); been sponsored for travel, registration or accommodation to attend conferences (61%).<br>- Percentage of patients who would like to know if their doctor obtains an indirect benefit/financial incentive for: instituting a course of

		treatment (80%); prescribing a drug (81%); making a referral (78%); doing a test or procedure (77%); enrolling patient in clinical trial (79%)<br>- 84% felt it is important for doctors to disclose any relevant competing interest.<br>- 78% believed that this disclosure would help patients to make better informed treatment decisions.<br>- Patients who would like their doctor to disclose his/her competing interests: verbally during the consultation (78%); by clearly displaying it on the wall of the consulting room (67%); by presenting it to them in a printed document (62%).<br>- 80% of patients stated that they would have more confidence in their doctor's decisions if interests were fully disclosed.
**Macneill, 2010[10]**<br><br>Supported by National Health and Medical Research Council of Australia.	• General public (over the age of 18 years) from the electoral roll of the Hunter region<br>• New South Wales, Australia<br>• N= 757; 59% female; average age (SD): 52.2 (±16.2)<br>• Education: 20% university degree or currently attending a university	**Attitudes towards the interactions:**<br>- The proportions of members of the public who 'always' or 'sometimes' considered it appropriate to accept each of the 'gifts' ranged from a low of 15% (for two movie tickets) to a high of 96% (for patient information leaflets on drugs).<br>- Overall, public respondents appeared to be more permissive about doctors accepting 'gifts' from pharmaceutical companies than do medical specialist respondents.<br>- Neither medical specialists nor members of the public were supportive of any 'gifts' that were clearly not relevant to medicine (laptop computer, tickets to theatre, sporting events) even when the cost of these was minimal.
**Grande, 2012[11]**<br><br>Funded by National Human Genome Research Institute, American Cancer Society	• Adults in 10 large metropolitan areas (random sample).<br>• USA; June -December 2006<br>• N= 2,029; 63.2% female; age: 8.4% aged 18-39, 62.2% aged 40-64, 29.4% aged 65 and above;<br>• Education: 28.2% some college; 35.3% 4-year college degree or graduate school. *(weighted %)	**Awareness of the interactions of own physicians:**<br>- 55% believed their personal physician accepts gifts from the pharmaceutical industry.<br>**Awareness of the interactions of physicians in general:**<br>- Patients who believed that the following proportion of doctors accept pharmaceutical industry gifts: all doctors (34%); some doctors (41%); almost no doctors (23%).<br>**Attitudes towards the effects of interactions on trust:**<br>- Participants who believed that physicians accept pharmaceutical industry gifts were more likely to report high health care system distrust compared to those that believed almost no doctors accept gifts.
**Green, 2012[12]**<br><br>Funding not reported	• English-speaking adults in outpatient clinics waiting rooms<br>• USA; 2008<br>• N= 192; 61% female; mean age (range): 53 (18–89);<br>• Education: 45% high school graduate or some college; 46% college graduate or more	**Awareness of the interactions of own physicians:**<br>- Respondents' knowledge of whether or not their physicians engage in the following activities with pharmaceutical companies: accept gifts over $100 (12%); attend drug companies' social activities (16%); attend industry-sponsored trips (17%); accept gifts less than $100 (16%); gave lectures (20%); conduct research for drug companies (23%); accept industry-sponsored meals (22%).<br>**Beliefs about their effects on prescription behavior and quality of care:**<br>- 43% believed that physicians who accepted small gifts in return for listening to an industry presentation on a particular medication would be more likely to prescribe that medication.<br>- 49% believed that accepting small gifts or meals would influence their physician's prescriptive behavior.<br>**Attitudes towards the interactions:**<br>- 24% said they would be less likely to take the prescribed medication if the physician had recently accepted a gift in return for listening to an industry presentation on that particular medication.<br>**Attitudes towards the effects of interactions on trust:**<br>- The percentage of respondents who indicated that their trust would be lower if physicians engage in the following activities: accepting gifts >$100 in value (59%); attending industry-sponsored trips (58%) and sporting events (54%); holding stock in companies producing medications prescribed by the physician (49%); accepting gifts of <$100 value (47%); giving lectures to drug companies (40%); accepting

		drug companies meal (33%); conducting research for drug companies (27%); using drug companies pens or notepads (5%).<br>- 43% indicated it was OK for physicians to accept small gifts or meals as long as gifts had little monetary value.<br>- In comparison to other professions, physicians felt it was "less wrong" for doctors to accept gifts from drug company representatives than it was for judges, lawyers, sport referees.<br>**Attitudes towards possible ways to manage the interactions:**<br>- A minority of respondents wanted to know whether their doctor accepts gifts and as the value of the gift increased, the percentage indicating it would negatively impact trust in their physician increased.<br>- Percentages of respondents who wanted to know whether their physician: accepted gifts > $100 in value (51%); attended drug companies social events (46%); went on trips paid by drug companies (43%); accepted gifts less than $100 (36%); gave lecture for drug companies (36%); conducted research for drug companies (35%); accepted drug companies meals (25%); used drug company pens or notepads (1%)
**Wise, 2012[13]**	• Postoperative South African patients from four surgical wards in a teaching hospital<br>• South Africa; March- November 2011<br>• N= 200; 67% females; age: 17% aged 18-24, 73% aged 25-64, 10% 65 or above<br>• Education: Not reported	**Beliefs about their effects on prescriptive behavior and quality of care:**<br>- 80% believed that doctors were influenced by gifts from the pharmaceutical company.<br>**Attitudes towards the interactions:**<br>- 81% preferred a physician who had no relationship with, or who did not accept gifts from, pharmaceutical companies.<br>- Participants who think doctors should be allowed to receive: free attendance at conferences and education classes (56%); free drug or medicine samples (46%); small gifts (38%); fees for speaking at conferences that are sponsored by pharmaceutical companies (21%); free food and dinners (12%); travel or holidays as gifts (2%).<br>**Attitudes towards the effects of interactions on trust:**<br>81% of postoperative patients preferred a physician who had no relationship with, or who did not accept gifts from, pharmaceutical companies.<br>**Attitudes towards possible ways to manage the interactions:**<br>- 66% felt that it was important to know about their physician's financial relationship with a pharmaceutical company.
**Camp, 2013[14]**<br><br>No external funding sources	• Postoperative arthroplasty patients attending follow up hip and knee arthroplasty clinics<br>• USA and Canada; November 2010 to March 2011<br>• N= 503; 55% females for US; 59% females for Canada; age: 36% less than 60, 64% 60 and above for US; 30% less than 60, 69% 60 and above for Canada<br>• Education: US (51% some college or university degree, 30% graduate or professional degree); Canadians (51% some college or university degree, 20% graduate or professional degree)	**Awareness of the interactions of surgeons in general:**<br>- 70% and 55% respectively of U.S. and Canadian patients were aware that physicians could have financial relationships with pharmaceutical companies

**Holbrook, 2013[15]**<br><br>Funded by Canadian Institutes of Health Research	• Adult population (18 years of age or older) who speak English or French and reside in private homes<br>• Canada; May-September 2010<br>• N= 1041; 56.8% female; mean age (SD): 52.6 (16.5);<br>• Education: 57.7% college or higher	**Attitudes towards the interactions:**<br>Respondents who approve of the following interactions between physicians and the pharmaceutical industry: calling a drug company for free information on one of their products (91.2%); receiving free samples for use among patients (78.7%); poster of heart with manufacturer's logo (77.6%); $100 for a 30-min talk about the company's drugs (21.7%); $100 for a 30-min talk about the company's drugs(9.6%); small gifts (54.9%); free dinner for educational purposes (54.5%); all-paid expenses to attend medical conferences (49.7%); free lunch for everyone who works in a medical office to talk about the company's drugs(49%); free samples for physician's personal use (22.5%); $100 per patient to recruit patients into drug company research studies (9%); $1000 per patient to recruit patients into drug company research studies (5%); physicians using information not yet publicly available about a promising new drug to make investment decisions (15.7%).
**Oakes, 2015[16]**<br><br>Funding not reported	• Patients (older than 18 years of age) from three of the academic health center's clinics (orthopedic surgery, cardiology and dentistry)<br>• USA, Twin Cities area Minnesota; nine-week period (no data)<br>• N=31 (a total of six focus groups); 74% female; mean age 55;<br>• Education: 65% college	**Beliefs about the interaction:**<br>Most participants expressed "cynical" views about the link between conflict of interest and physician behavior.<br>**Attitudes towards the interaction:**<br>-Few participants expressed concern about the topic itself and felt that physicians and other health care providers always acted in the patient's best interest.<br>**Attitudes towards the effects of interactions on trust:**<br>-There was near unanimous agreement that when clinicians did not voluntarily disclose a conflict of interest when one existed, they put their relationships with patients in jeopardy.<br>**Attitudes towards possible ways to manage the interactions:**<br>-None of the participants thought clinics should post their doctors' conflicts of interest on clinic websites or on signs.<br>-The majority of participants did not support idea of a mailed letter regarding a conflict of interest<br>-Most participants wanted the information about the conflict of interest presented in a simple-to-read paper document during clinic check-ins. A key point was that the disclosure form should list contact information for persons with knowledge of the issues so that patients could follow up if they wished.<br>-The most frequently expressed opinion was that a physician should bring up the subject of a potential conflict of interest to a patient only if such disclosure was directly relevant to a specific aspect of their treatment orrcare.<br>-The primary concern with verbal disclosure was that discussion about conflict of interest would distract from patient care and/or use up valuable visit time.

References

1. Blake RL, Jr., Early EK: **Patients' attitudes about gifts to physicians from pharmaceutical companies**. *The Journal of the American Board of Family Practice / American Board of Family Practice* 1995, **8**(6):457-464.
2. La Puma J, Stocking CB, Rhoades WD, Darling CM, Ferner RE, Neuberger J, VandenBurg M, Dews I, Tobias JS: **Financial ties as part of informed consent to postmarketing research. Attitudes of American doctors and patients**. *Bmj* 1995, **310**(6995):1660-1663.
3. Mainous AG, 3rd, Hueston WJ, Rich EC: **Patient perceptions of physician acceptance of gifts from the pharmaceutical industry**. *Archives of family medicine* 1995, **4**(4):335-339.
4. Gibbons RV, Landry FJ, Blouch DL, Jones DL, Williams FK, Lucey CR, Kroenke K: **A comparison of physicians' and patients' attitudes toward pharmaceutical industry gifts**. *Journal of general internal medicine* 1998, **13**(3):151-154.
5. Qidawai WQ, H.; Sohail, S.; Alam, M.; Azam, S.: **PERCEPTIONS ON BIOETHICS AMONG PATIENTS PRESENTING TO FAMILY PHYSICIANS AT A TEACHING HOSPITAL IN KARACHI, PAKISTAN**. *Pak J Med Sci* 2003, **19**(3):192-196.
6. Semin S, Guldal D, Ozcakar N, Mevsim V: **What patients think about promotional activities of pharmaceutical companies in Turkey**. *Pharmacy world & science : PWS* 2006, **28**(4):199-206.
7. Edwards D, Ballantyne A: **Patient awareness and concern regarding pharmaceutical manufacturer interactions with doctors**. *Internal medicine journal* 2009, **39**(3):191-196.
8. Jastifer J, Roberts S: **Patients' awareness of and attitudes toward gifts from pharmaceutical companies to physicians**. *International journal of health services : planning, administration, evaluation* 2009, **39**(2):405-414.
9. Tattersall MH, Dimoska A, Gan K: **Patients expect transparency in doctors' relationships with the pharmaceutical industry**. *The Medical journal of Australia* 2009, **190**(2):65-68.
10. Macneill PU, Kerridge IH, Newby D, Stokes BJ, Doran E, Henry DA: **Attitudes of physicians and public to pharmaceutical industry 'gifts'**. *Internal medicine journal* 2010, **40**(5):335-341.
11. Grande D, Shea JA, Armstrong K: **Pharmaceutical industry gifts to physicians: patient beliefs and trust in physicians and the health care system**. *Journal of general internal medicine* 2012, **27**(3):274-279.
12. Green MJ, Masters R, James B, Simmons B, Lehman E: **Do gifts from the pharmaceutical industry affect trust in physicians?** *Family medicine* 2012, **44**(5):325-331.
13. R. WRR: **The opinion of patients at a local South African teaching hospital on physician - industry relations**. *South Afr J Anaesth Analg* 2013, **19**(3):160-163.
14. Camp MW, Mattingly DA, Gross AE, Nousiainen MT, Alman BA, McKneally MF: **Patients' views on surgeons' financial conflicts of interest**. *The Journal of bone and joint surgery American volume* 2013, **95**(2):e9 1-8.
15. Holbrook A, Lexchin J, Pullenayegum E, Campbell C, Marlow B, Troyan S, Weijer C, Blackmer J, Brazil K, Willison D: **What do Canadians think about physician-pharmaceutical industry interactions?** *Health policy* 2013, **112**(3):255-263.
16. Oakes JM, Whitham HK, Spaulding AB, Zentner LA, Beccard SR: **How should doctors disclose conflicts of interest to patients? A focus group investigation**. *Minnesota medicine* 2015, **98**(1):38-41.
