# Supplementary material for: Knowledge, Beliefs and Attitudes of Patients and the General Public towards the Interactions of Physicians with the Pharmaceutical and the Device Industry: A Systematic Review
Source: PLoS One. 2016 Aug 24;11(8):e0160540. doi: 10.1371/journal.pone.0160540 (PMC4996522; doi:10.1371/journal.pone.0160540)
Supplement: S3 Table — (PDF) [file pone.0160540.s006.pdf]

S3 Table. Methodological features of included studies on interactions with device industry

Study ID	Sample size calculation	Sampling:<br>•Frame<br>•Method (type)	Recruitment method	Respons e rate	Administration method	Validity of tool<br>Pilot testing done
Khan, 2007[1]	Not reported	•Frame: Patients in the waiting area in orthopedic surgery clinic<br>•Method: Convenient sampling .”	In person	Not reported	In person, self-administered	•Validity of tool: Not reported<br>•Pilot testing: Not reported.
Fisher, 2012[2]	Not reported	•Frame: North American public visiting the spineuniverse.com website.<br>•Method: convenient sampling “The survey was posted on www.spineuniverse. com […] All respondents voluntarily filled out the survey”	Internet- unrestricted self-selected survey	Not reported: not applicabl e	Internet “The survey was posted on www.spineuniverse.com”	•Validity of tool: Partially validated “Multiple iterations of the survey were generated to ensure question clarity and inclusion of relevant content.”<br>•Pilot-testing: Yes “Each rendition was piloted on small samples of the general population
Camp, 2013[3]	Not reported	•Frame: postoperative patients attending follow up hip and knee arthroplasty clinics at Mount Sinai Hospital and Holland Orthopaedic , Arthritic Centre and the New England Baptist Hospital in US(1) and Canada (2) English-speaking patients who were at least eighteen years old and who had undergone primary or revision hip or knee arthroplasty at least three months earlier were eligible to participate in the study.<br>•Method: Convenient sampling	In person- “Patients attending follow-up arthroplasty clinics at the participating hospitals were asked by clinic personnel or the first author”	88% for US, 92% for Canada, Combine d rate: 90%	In-person, self-administered questionnaires (with no assistance)<br><br>40 item questionnaire (3-likert scale):	•Validity of tool: Not reported<br>•Pilot testing: Yes “Testing the derived questionnaire on nonsurgical volunteers and postoperative arthroplasty patients in addition to using cognitive interviews to ensure that questions would be understood.”

Lieberman, 2013[4]	No	•Frame: All patients 18 years old or older scheduled for primary THA and TKA from the orthopedic practices of two joint arthroplasty specialists at an academic health center.<br>•Method: convenient sampling	In person<br>"Just before the 6-week follow-up visit, the surgeons asked the patients if they wanted to participate in this study by filling out a survey"	41%	In person, self-administered questionnaire	•Validity of tool: Self-developed tool, no validation reported<br>•Pilot testing: not reported.
Dipaola, 2014[5]	No	•Frame: visitors of the spineuniverse.com website.<br>•Method: convenient sampling<br>"The survey was posted on http://www.spineuniverse. com/ […] All respondents voluntarily filled out the survey in a Web-based format posted on http://www.spineuniverse. com/."	Internet - unrestricted self-selected survey	Not reported: not applicable	Internet<br>"Survey was administered using a ''spine Web site"	•Validity of tool: Partial validation<br>"Multiple iterations of the survey were generated to ensure that the questions were clear and included relevant content"<br>•Pilot testing: Yes.<br>"Each rendition was piloted on small samples of the general population.""

References

1. Khan MH, Lee JY, Rihn JA, Cassinelli EH, Lim MR, Kang JD, Donaldson WF, 3rd: **The surgeon as a consultant for medical device manufacturers: what do our patients think?** *Spine* 2007, **32**(23):2616-2618; discussion 2619.
2. Fisher CG, DiPaola CP, Noonan VK, Bailey C, Dvorak MF: **Physician-industry conflict of interest: public opinion regarding industry-sponsored research**. *Journal of neurosurgery Spine* 2012, **17**(1):1-10.
3. Camp MW, Mattingly DA, Gross AE, Nousiainen MT, Alman BA, McKneally MF: **Patients' views on surgeons' financial conflicts of interest**. *The Journal of bone and joint surgery American volume* 2013, **95**(2):e9 1-8.
4. Lieberman JR, Pensak MJ, Kelleher MS, Leger RR, Polkowski GG, 2nd: **Disclosure of financial conflicts of interest: an evaluation of orthopaedic surgery patients' understanding**. *Clinical orthopaedics and related research* 2013, **471**(2):472-477.
5. DiPaola CP, Dea N, Noonan VK, Bailey CS, Dvorak MF, Fisher CG: **Surgeon-industry conflict of interest: survey of North Americans' opinions regarding surgeons consulting with industry**. *The spine journal : official journal of the North American Spine Society* 2014, **14**(4):584-591.
