# Supplementary material for: Knowledge, Beliefs and Attitudes of Patients and the General Public towards the Interactions of Physicians with the Pharmaceutical and the Device Industry: A Systematic Review
Source: PLoS One. 2016 Aug 24;11(8):e0160540. doi: 10.1371/journal.pone.0160540 (PMC4996522; doi:10.1371/journal.pone.0160540)
Supplement: S4 Table — (PDF) [file pone.0160540.s007.pdf]

S4 Table: Summary of findings on interactions with device industry

Study ID/	Participants and settings	Results
**Khan, 2007[1]**<br><br>Funding not reported	• Patients in the waiting area in orthopedic surgery clinic<br>• USA<br>• N = 245; 51.0% female; average age: 55.5 (±14.5)<br>• Education: 33.9% college graduates; 19.2% graduate/postgraduates	**Attitudes towards the interactions:**<br>- 94.3% thought doctors' advisory role towards medical device manufacturers to would be beneficial to patients.<br>- 66.5% believed that physicians should be compensated for this advisory role.<br>- 72.7% wanted to know if the device being recommended by their physician was actually designed by him/her.<br>- 89.4% believed that physicians should be allowed to recommend the use of a device that they helped design.<br>**Attitudes towards possible ways to manage the interactions**:<br>- 48.2% thought that physician-medical device manufacturer relationship should be regulated.<br>- Entity that should be involved in regulating physician-medical device manufacturer relationship: physicians (32.2%); hospitals (20%); government (13.5%); no answer (34.3%).
**Fisher, 2012[2]**<br><br>Funding not reported	• North American public visiting the spineuniverse.com website<br>• USA; 2 weeks (date not reported)<br>• N=501; 63.3% females; 46.9% aged 30-49, 26.1% aged 50-59, and 19.2% aged 60 and above;<br>• Education: 52% tech or 4-year college; 25.7% graduate	**Beliefs about their effects on quality of care:**<br>- 55% believed that the source of medical research funding for a study would affect the quality of their care.<br>**Attitudes towards the interactions:**<br>- 91% felt that surgeon input is important for industry-funded research.<br>- 67% felt that industry-funded and government-funded research could be equally honest and objective.<br>**Attitudes towards possible ways to manage the interactions:**<br>- 69% felt that surgeons should be allowed to perform research on products in which they have a financial interest as long as guidelines are set up to regulate potential conflict of interest.<br>- 71.7% of respondents believe that a combination of the following entities should be involved in regulating surgeon-industry consulting relationship: government, hospitals or universities, medical company representatives, and medical professional societies. 34.1% stated that medical professional societies should have the most power in this regulation (43.3% not sure).<br>- 30.4% think that medical company representatives should not be involved in regulating surgeon-industry consulting relationships (but 42.1% were not sure).
**Camp, 2013[3]**<br><br>No external funding sources	• Postoperative arthroplasty patients attending follow up hip and knee arthroplasty clinics<br>• USA and Canada; November 2010 to March 2011<br>• N= 503; 55% females for US; 59% females for Canada; age: 36% less than 60, 64% 60 and above for US; 30% less than 60, 69% 60 and above for Canada<br>• Education: US (51% some college or university degree, 30% graduate or professional degree); Canadians (51% some college or university degree, 20%	**Awareness of the interactions of surgeons in general:**<br>- 54% and 35% respectively of U.S. and Canadian patients were aware that: surgeons could have financial relationships with device manufacturers<br>**Attitudes towards the interactions:**<br>- Percentages of U.S and Canadian patients, respectively, who were worried about possible financial relationships between: their surgeon and industry (6% versus 6%); their surgeon and manufacturers (17% versus 22%).<br>- U.S. patients and Canadian patients, respectively, who thought it was appropriate for their surgeon to: receive royalties for a patent on a product that the surgeon had designed (69% versus 66%); receive payments for offering advice to the company in their area of expertise (48% versus 53%); receive payments to give lectures on the company's products (46% versus 53%); receive gifts from industry worth more than $100 (11% versus 13%); receive gifts from industry worth less $100 from a company (20% versus 18%); own shares in company that supplied their prosthesis (21% versus 22%)<br>**Beliefs about their effects on quality of care:**<br>-76% of U.S. patients and 74% of Canadian patients felt their surgeon would make the best choices for their health, regardless of

	graduate or professional degree)	financial relationships with device manufacturers.<br>**<u>Attitudes towards possible ways to manage the interactions:</u>**<br>- 47% and 42% of patients in the U.S. and in Canada respectively wanted their surgeon to verbally disclose financial relationships with manufacturers. 42% and 38% of patients in the U.S. and in Canada respectively wanted this disclosure in the form of a pamphlet.<br>- 38% of U.S. patients and 30% of Canadian patients agreed that surgeons should place their financial relationships on a publicly accessible web site.<br>- US patients and Canadian patients, respectively, who wanted financial relationships to be regulated by: their surgeon's professional organization (83% versus 83%); their surgeon (81% versus 78%); a new committee at their surgeon's hospital (60% versus 61%); a government agency (26% versus 35%).
**Lieberman, 2013[4]**<br><br>Funding from the NIH Musculoskeletal Transplant Foundation	• Patients (18 years old or older) scheduled for primary THA and TKA from the orthopedic practices of two joint arthroplasty specialists<br>• USA; September 2010 to September 2011<br>• N= 100; 66% female; mean age (SD): 63 (±13.3)<br>• Education: 49% college; 20% Master's or Doctoral degree	**<u>Awareness of the interactions of surgeons in general:</u>**<br>- 47% were aware of financial conflict of interests related to clinical research.<br>- 13% had already received information regarding financial conflict of interests from a surgeon.<br>**<u>Attitudes towards the interactions:</u>**<br>- Respondents who were either not concerned or minimally concerned about: their surgeon's potential FCOI (81%); institutional FCOI (79%).<br>- 51% indicated that if their surgeon had developed a prosthesis used in their surgery, they would be more willing to have this surgeon perform the operation (43% were neutral).<br>- Respondents who would be less likely to be operated on by surgeons if: they had developed a prosthesis used in their surgery (14%); they received revenue from a company (40%); if he or she has a stock in a company (44%).<br>- 43% of patients were concerned if the surgeon was paid by a company that manufactured a product used in surgery.<br>**<u>Beliefs about their effects on trust:</u>**<br>- 24% indicated they would trust a surgeon less if he or she had FCOI (44% disagreed).<br>**<u>Attitudes towards possible ways to manage the interactions:</u>**<br>- 55% believed that surgeons should make patients aware of FCOI.
**Dipaola, 2014[5]**<br><br>Funding not reported	• North Americans representing the general public visiting the spineuniverse.com website<br>• USA; 2 weeks (no data)<br>• N= 610; 63.3% females; 42.8% aged 30-49, 31% aged 50-59, 21% aged> 60<br>• Education: 54.8% technical school college; 24.6% graduate school	**<u>Attitudes towards the interactions:</u>**<br>- 82% felt it is ethical for surgeons to work with companies as consultants to design/improve health-care products/devices<br>**<u>Beliefs about their effects on quality of care:</u>**<br>- Respondents who believed that their care will be worse if: their surgeon is a consultant to help design/improve a surgical device (19.5%); royalties are paid to the surgeon when he/she uses the product (39.2%); royalties are paid to the surgeon only for devices that other surgeons use (24.6%),<br>**<u>Attitudes towards possible ways to manage the interactions:</u>**<br>- Respondents who felt that their surgeons should disclose consulting relationships: to all patients (61.6%); only to patients receiving the device (91.1%).<br>- 64.3% believe that a combination of entities including government, hospitals, universities, medical company representatives, and medical professional societies should be involved in regulating surgeon-industry consulting relationship, with 34.9% stating that medical professional societies should have the most power in this regulation.<br>- 44.9% think that medical company representatives should not be involved in regulating surgeon-industry consulting relationships (the majority were not sure).

References

1. Khan MH, Lee JY, Rihn JA, Cassinelli EH, Lim MR, Kang JD, Donaldson WF, 3rd: **The surgeon as a consultant for medical device manufacturers: what do our patients think?** *Spine* 2007, **32**(23):2616-2618; discussion 2619.
2. Fisher CG, DiPaola CP, Noonan VK, Bailey C, Dvorak MF: **Physician-industry conflict of interest: public opinion regarding industry-sponsored research**. *Journal of neurosurgery Spine* 2012, **17**(1):1-10.
3. Camp MW, Mattingly DA, Gross AE, Nousiainen MT, Alman BA, McKneally MF: **Patients' views on surgeons' financial conflicts of interest**. *The Journal of bone and joint surgery American volume* 2013, **95**(2):e9 1-8.
4. Lieberman JR, Pensak MJ, Kelleher MS, Leger RR, Polkowski GG, 2nd: **Disclosure of financial conflicts of interest: an evaluation of orthopaedic surgery patients' understanding**. *Clinical orthopaedics and related research* 2013, **471**(2):472-477.
5. DiPaola CP, Dea N, Noonan VK, Bailey CS, Dvorak MF, Fisher CG: **Surgeon-industry conflict of interest: survey of North Americans' opinions regarding surgeons consulting with industry**. *The spine journal : official journal of the North American Spine Society* 2014, **14**(4):584-591.
